# Deciphering the roles of subcellular distribution and interactions involving the MEF2 binding region, the ankyrin repeat binding motif and the catalytic site of HDAC4 in *Drosophila* neuronal morphogenesis

**DOI:** 10.1186/s12915-023-01800-1

**Published:** 2024-01-02

**Authors:** Wei Jun Tan, Hannah R. Hawley, Sarah J. Wilson, Helen L. Fitzsimons

**Affiliations:** School of Natural Sciences, Massey University, Palmerston North, New Zealand

**Keywords:** Drosophila, HDAC4, Histone deacetylase, Brain, Neuron, Mushroom body, Eye, Photoreceptor, MEF2, Ankyrin repeats

## Abstract

**Background:**

Dysregulation of nucleocytoplasmic shuttling of histone deacetylase 4 (HDAC4) is associated with several neurodevelopmental and neurodegenerative disorders. Consequently, understanding the roles of nuclear and cytoplasmic HDAC4 along with the mechanisms that regulate nuclear entry and exit is an area of concerted effort. Efficient nuclear entry is dependent on binding of the transcription factor MEF2, as mutations in the MEF2 binding region result in cytoplasmic accumulation of HDAC4. It is well established that nuclear exit and cytoplasmic retention are dependent on 14–3-3-binding, and mutations that affect binding are widely used to induce nuclear accumulation of HDAC4. While regulation of HDAC4 shuttling is clearly important, there is a gap in understanding of how the nuclear and cytoplasmic distribution of HDAC4 impacts its function. Furthermore, it is unclear whether other features of the protein including the catalytic site, the MEF2-binding region and/or the ankyrin repeat binding motif influence the distribution and/or activity of HDAC4 in neurons. Since HDAC4 functions are conserved in *Drosophila*, and increased nuclear accumulation of HDAC4 also results in impaired neurodevelopment, we used *Drosophila* as a genetic model for investigation of HDAC4 function.

**Results:**

Here we have generated a series of mutants for functional dissection of HDAC4 via in-depth examination of the resulting subcellular distribution and nuclear aggregation, and correlate these with developmental phenotypes resulting from their expression in well-established models of neuronal morphogenesis of the *Drosophila* mushroom body and eye. We found that in the mushroom body, forced sequestration of HDAC4 in the nucleus or the cytoplasm resulted in defects in axon morphogenesis. The actions of HDAC4 that resulted in impaired development were dependent on the MEF2 binding region, modulated by the ankyrin repeat binding motif, and largely independent of an intact catalytic site. In contrast, disruption to eye development was largely independent of MEF2 binding but mutation of the catalytic site significantly reduced the phenotype, indicating that HDAC4 acts in a neuronal-subtype-specific manner.

**Conclusions:**

We found that the impairments to mushroom body and eye development resulting from nuclear accumulation of HDAC4 were exacerbated by mutation of the ankyrin repeat binding motif, whereas there was a differing requirement for the MEF2 binding site and an intact catalytic site. It will be of importance to determine the binding partners of HDAC4 in nuclear aggregates and in the cytoplasm of these tissues to further understand its mechanisms of action.

**Supplementary Information:**

The online version contains supplementary material available at 10.1186/s12915-023-01800-1.

## Background

Histone deacetylases (HDACs) are a family of epigenetic regulators that influence neuronal development, survival and function. HDAC4 is a Class IIa HDAC, a subgroup of HDACs characterized by their extended N-terminal region that contains binding sites for various transcriptional regulators [1–4]. Although Class IIa HDACs are highly conserved among metazoans [5, 6], vertebrate Class IIa HDACs display weak activity on canonical acetylated substrates due to a H976Y amino acid substitution within the active site [6]. HDAC4-dependent deacetylation is facilitated via association with HDAC3 as part of the NcoR/SMRT repressor complex [7–9], and while originally characterized as histone deacetylases, Class II HDACs can also facilitate deacetylation of non-histone targets [10, 11]. Notably, class IIa HDACs undergo nucleocytoplasmic shuttling rather than exclusively residing in the nucleus, with their nuclear entry and exit mediated by nuclear localization and export signals, respectively [4]; however, binding of additional factors is required to facilitate dynamic activity-dependent regulation of shuttling. In neurons, nuclear export is initiated in response to synaptic activity by activation of Ca^2+^/calmodulin-dependent protein kinases [12] that phosphorylate serine residues S246, S467 and S632 to create a docking site for the chaperone 14–3-3, which exports HDAC4 from the nucleus [4, 13–18]. In addition, S246 is adjacent to the nuclear localization signal (NLS), and binding of 14–3-3 blocks this site, inhibiting association of importin-α and therefore sequestering HDAC4 in the cytoplasm [15, 17]. Conversely, dephosphorylation by the serine/threonine phosphatase PP2A allows nuclear re-entry [19]. Mutation of the three serine residues prevents 14–3-3-mediated exit, resulting in permanent sequestration of HDAC4 in the nucleus. Efficient nuclear entry is also dependent on binding of the transcription factor MEF2, as mutations in the MEF2 binding region result in cytoplasmic accumulation [4, 20].

Total levels of HDAC4 are elevated in brains of individuals with autism [21] as well as in animal models of Alzheimer’s disease [22, 23], and depression [24, 25]. Dysregulation of nucleocytoplasmic shuttling is also associated with several neurodevelopmental and neurodegenerative disorders. Increased nuclear accumulation of HDAC4 has been observed in the postmortem brains of individuals with Alzheimer’s disease [26], as well as in neuronal nuclei of Alzheimer’s disease [26], ataxia telangiectasia [27] and Parkinson’s disease [28] models. Any disruption to shuttling that results in nuclear accumulation must also involve cytoplasmic depletion, and reduced expression of HDAC4 is also associated with neurodevelopmental deficits. Heterozygous deletion of 2q37, a chromosomal region including *HDAC4*, is associated with neurological features including developmental delay, intellectual disability and autism [29–32]. While the loss of *HDAC4* has been attributed as the main cause [33], the haploinsufficiency phenotype displays incomplete penetrance, suggesting contribution from other genes [33–35]. Several cases have been identified however, that involve de novo heterozygous mutations predicted to result in dysregulation of HDAC4 subcellular shuttling. One such case involves a single-nucleotide insertion that results in a frameshift and truncation of the C-terminal domain of HDAC4, producing a truncated protein lacking the deacetylase domain and nuclear export sequence (NES) [31]. When expressed in cultured neurons, this truncated form of HDAC4 was sequestered in the nucleus, and its expression in the mouse forebrain resulted in impaired learning and memory [36]. Five individuals sharing similar clinical features including developmental delay, hypotonia and intellectual disability were all identified to carry de novo heterozygous missense substitutions in HDAC4 14–3-3 binding sites predicted to result in its nuclear translocation [37]. This points strongly towards disruption of HDAC4 nucleocytoplasmic shuttling as the underlying cause. Disruption to the subcellular regulation of HDAC4 has also been implicated in CDKL5 disorder, in which mutations in *cyclin dependent kinase-like 5* (*Cdkl5)* [38] cause severe intellectual disability and motor impairment [39]. When modelled in *CDKL5* knockout mice, increased nuclear retention of HDAC4 was observed, which correlated with impaired synapse development and hippocampal-dependent learning and memory. However, this was reversed by treatment with the HDAC4/5 inhibitor LMK235 [38].

Dysregulation of HDAC4 also results in impaired neurodevelopment in *Drosophila*. Human and *Drosophila* HDAC4 share 35% identity and 59% similarity overall, with 57% amino acid identity and 84% similarity through the C-terminal deacetylase domain [40]. Furthermore, HDAC4 is the sole Class IIa HDAC in this species; therefore, study of HDAC4 in *Drosophila* can uncover features hidden by redundancy of the four vertebrate HDACs. We previously demonstrated defects in development of the *Drosophila* mushroom body upon altered regulation of HDAC4 [41]. The *Drosophila* mushroom body is a paired neuropil structure that is ideal for studying neurodevelopmental processes as the axons of the Kenyon cells (the intrinsic neurons of the mushroom body) are bundled together into densely packed fibers. This allows any defects in axon morphogenesis such as elongation, guidance and termination to be easily visualized and quantified [42–44]. Increased expression of *Drosophila* HDAC4 or ectopic expression of human HDAC4 results in impaired elongation and termination of mushroom body axons [41]. Similarly, increased expression of *Drosophila* or human HDAC4 also disrupts development of the *Drosophila* eye [41, 45], which consists of a highly organized lattice of 800 ommatidia [46]. The pathways involved in eye development are increasingly well understood [47, 48] and disruption to development is readily visualized and quantified [49, 50], thus also providing a valuable model for dissection of HDAC4 function in neurons.

To decipher the mechanisms through which HDAC4 impairs neuronal development in *Drosophila*, we previously carried out RNA-seq on heads of flies overexpressing *Drosophila* HDAC4 in the adult fly brain and found few significant transcriptional changes [45]. We reasoned that this may be due to a dilution effect, given that HDAC4 is nuclear in only a subset of cells. We therefore performed RNA-seq on heads of flies expressing nuclear-localized and cytoplasmically localized mutants of human HDAC4 in the adult brain. Only 29 genes were significantly differentially expressed between these mutants and only four of these genes displayed more than a log_2_-fold change in expression [41]. These data suggest that HDAC4 may act at least in part through non-transcriptional mechanisms; therefore, an increased understanding of its regulation may shed light on this.

As aforementioned, human and *Drosophil*a HDAC4 are highly conserved, including the NLS, NES and 14–3-3 binding sites, each of which are critical to regulation of HDAC4 distribution and function in neurons [12, 15, 17, 36, 41, 51]. The MEF2 binding region is also conserved, and HDAC4 has been observed to form aggregates that sequester MEF2 in neuronal nuclei [40, 41]. HDAC4 exists in dynamic equilibrium between monomeric, and multimeric species in vitro, with tetramerization mediated through the N-terminal alpha-helices of four HDAC4 molecules forming a four-helix bundle [52]. HDAC4 multimers are also observed in vivo where they have the capacity to form into higher-order structures which are commonly referred to as speckles or aggregates [2, 20, 40, 41, 53]; however, the importance of HDAC4 aggregation and subsequent sequestration of MEF2 to the manifestation of neurodevelopmental defects is currently not clear. The PxLPxI/L ankyrin repeat binding motif of HDAC4 is also conserved in *Drosophila* (PSLPNI in both *species*) [54]. This has been shown to mediate interaction of human HDAC4 with regulatory factor X associated ankyrin containing protein (RFXANK), a regulator of the major histocompatibility complex (MHC) class II genes, and ankyrin repeat family A member 2 (ANKRA2), a binding partner of megalin in epithelial cells, via this motif [54–56], but it is not yet known whether it plays an important role in HDAC4 function in neurons.

In summary, while regulation of HDAC4 shuttling is clearly important, there are still gaps in understanding of how the nuclear and cytoplasmic distribution of HDAC4 impacts its function. Moreover, it is unclear how other features of the protein including the deacetylase activity, MEF2-binding and/or the ankyrin repeat binding motif influence the distribution and/or activity of HDAC4 in neurons. To that end, here we sought to generate a panel of mutants to dissect the importance of subcellular distribution as well as structural features of HDAC4 in our well-established models of neuronal morphogenesis in the eye and brain.

## Results

### Expression and subcellular distribution of the HDAC4 mutants

**Fig.1:**
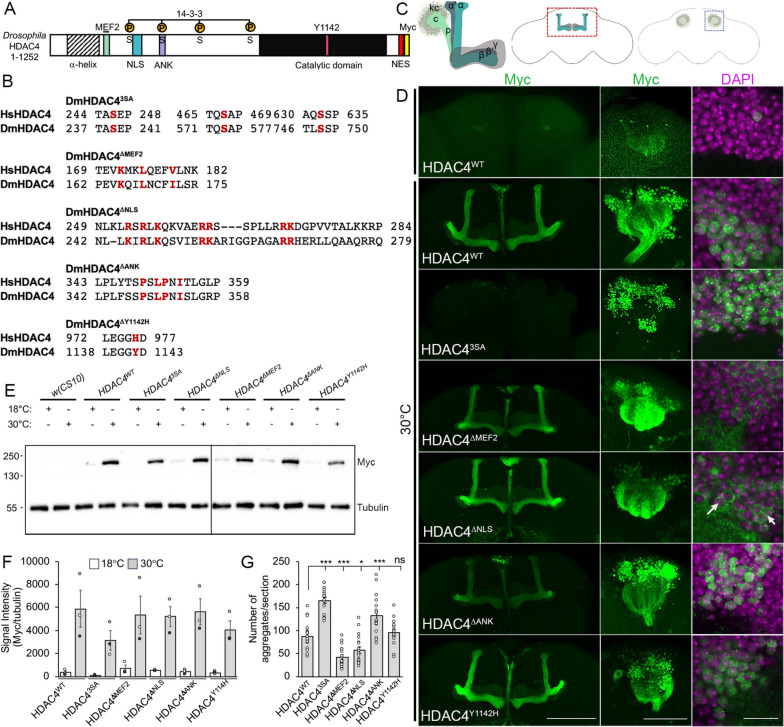
Expression and subcellular localization of HDAC4^WT^ and mutants. **A** Schematic of the domain structure of *Drosophila* HDAC4. Green, MEF2 binding site; blue, NLS; red, NES; purple, ankyrin repeat binding domain; yellow circles, phosphorylated serine residues that bind 14–3-3 proteins; black, catalytic domain; pink, location of the Y1142 residue in the catalytic site; yellow Myc tag. Shaded area depicts the α helix region important in tetramerization. **B** Amino acid substitutions present in each of the HDAC4 mutants are shown in red. All residues in red were mutated to alanines. **C** Cartoon of the mushroom body showing the Kenyon cells (kc), peduncle (p), calyx (c) and α, α', β, β' and γ lobes of the mushroom body. The location of the lobes in the anterior region of the brain (red square) and the Kenyon cells at the posterior (blue square) is also shown. **D**–**G** All genotypes were generated by crossing *OK107-GAL4; tubP-GAL80ts* females to males carrying the indicated *UAS-HDAC4* transgene. Flies were raised at 18 °C, at which temperature GAL80ts represses GAL4, until after eclosion when the temperature was raised to 30 °C. At this temperature, GAL80ts is inactivated, allowing GAL4 to induce transgene expression. Brains were dissected after 48 h at 30 °C. **D** Immunohistochemistry on whole mount brains with anti-Myc (green) counterstained with DAPI (magenta). Representative images from *n* = 16–19 brains per genotype are shown. The top panel shows uninduced HDAC4^WT^ and the panels below show expression of each of the transgenes following induction. The left panel shows a *Z*-stack maximum projection spanning the mushroom body lobes. Scale bar = 100 μm. The middle panel is a *Z*-stack through the Kenyon cell layer of the same brain. Scale bar = 100 μm. The right panel is a single 1 μm optical section through the cell bodies of the Kenyon cells Scale bar = 10 μm. Arrows indicate small puncta in HDAC4^ΔNLS^ nuclei that are substantially smaller than those in HDAC4^WT^ nuclei. **E** Western blot showing expression of each of the transgenes at 18 °C (uninduced) and 30 °C (induced) as detected by anti-Myc with α-tubulin as a loading control. Line through the center indicates joining of two blots that were processed simultaneously. **F** Quantification of Western blot. HDAC4^WT^ and mutant protein levels were normalized to tubulin. Error bars indicate SEM of blots from three independent fly crosses. There were no significant differences in protein levels at 18 °C (ANOVA F_(5,12)_ = 1.974, *p* = 0.156) or 30 °C (ANOVA, F_(5,12)_ = 0.787, *p* = 0.579). **G** Quantification of nuclear aggregates. The number of aggregates per section were counted from three sections per brain, *n* = 16–19 brains/genotype. The number of aggregates was significantly increased in the Kenyon cell nuclei of HDAC4^3SA^ and HDAC4^ΔANK^ brains and was significantly reduced in HDAC4^ΔMEF2^ and HDAC4^ΔNLS^ brains in comparison to HDAC4^WT^ (ANOVA, F_(5,97)_ = 38.46, *p* = 1.11 × 10.^−16^, Tukey’s post hoc test, *p < 0.05, ***p < 0.001)

To investigate the relative importance of these regions of the HDAC4 protein to its function, we generated *UAS-HDAC4* constructs carrying mutations in the MEF2 binding region (HDAC4^ΔMEF2^) [4], ankyrin repeat binding motif (HDAC4^ΔANK^) [54] and catalytic site (HDAC4^Y1142H^, equivalent to human H976Y) [6] (Fig. 1A, B). Disruption of MEF2 binding by HDAC4^ΔMEF2^ was confirmed by immunohistochemistry (Additional File 1). Since mutation of the MEF2 binding region results in cytoplasmic accumulation, to dissociate effects of MEF2 binding from subcellular distribution, we also generated a mutant with substitutions in conserved serine and arginine residues within the NLS (HDAC4^ΔNLS^), which are required for nuclear localization of human HDAC4 [4]. This was compared alongside a nuclear-restricted mutant with alanine substitutions of serine residues 239, 573 and 748, which are required for nuclear export of HDAC4 (HDAC4^3SA^) [12, 15, 17, 36, 41, 51].

We first characterized the expression patterns in the mushroom body. The intrinsic neurons of the mushroom body are the Kenyon cells, which comprise three subtypes; α/β, α′/β′ and γ (Fig. 1C). The cell bodies and dendrites are clustered at the posterior of the brain and their axons project ventrally in a bundled fiber termed the peduncle, before separating into lobes. The α/β and α′/β′ axons both bifurcate to form the vertical α and α′ lobes and the medial β and β′ lobes, while the γ axons form a single medial lobe [44]. The HDAC4^WT^ and mutant transgenes were expressed via the UAS/GAL4 system [57] with the *OK107-GAL4* driver, which drives strong expression in all Kenyon cell subtypes [58, 59]. To avoid developmental defects that may disrupt axon morphogenesis and thus complicate visualization of subcellular distribution, expression was restricted to the adult brain via co-expression of temperature-sensitive GAL80 (GAL80ts). At 18 °C, GAL80ts binds and inhibits GAL4 activity to prevent transgene expression [60]. Following eclosion, flies were incubated at 30 °C to inactivate GAL80ts and thus induce expression. HDAC4^WT^ was distributed through the entire mushroom body, with protein detected in nuclei, the calyx (dendritic field), peduncle and lobes (Fig. 1D). Within a subset of nuclei, HDAC4^WT^ aggregated into nuclear puncta, an expression pattern that was observed previously for FLAG- and GFP-tagged *Drosophila* and human HDAC4 [40, 41]. As expected, HDAC4^3SA^ was restricted to nuclei where it formed large aggregates and was completely absent from lobes. HDAC4^ΔMEF2^ and HDAC4^ΔNLS^ were almost completely excluded from nuclei, while showing strong localization to the lobes. Very small nuclear aggregates were sometimes observed in HDAC4^ΔMEF2^ and HDAC4^ΔNLS^, although these were less frequent in HDAC4^ΔMEF2^ expressing neurons. HDAC4^ΔANK^ also displayed a shift in distribution to more a nuclear accumulation, however not to the extent of HDAC4^3SA^. The expression pattern of HDAC4^Y1142H^ appeared indistinguishable from that of HDAC4^WT^. There was no significant difference in the expression levels of any of the mutants in whole-cell lysates (Fig. 1E, F), although we have consistently detected HDAC4^3SA^ at a lower level, which may be a consequence of less efficient extraction from nuclei where HDAC4^3SA^ is sequestered. To quantify any changes in nuclear accumulation from that of HDAC4^WT^, the number of nuclear aggregates in Kenyon cells was quantified (Fig. 1G), which confirmed the distribution patterns as observed via immunohistochemistry. Notably there were twice as many nuclear aggregates present in cells expressing HDAC4^3SA^ when compared to HDAC4^WT^. HDAC4^3SA^ and HDAC4^ΔANK^ aggregates were increased in size compared to HDAC4^WT^, and it is hypothesized that this is a result of coalescence of smaller aggregates. The reverse was observed for HDAC4^ΔNLS^ and HDAC4^ΔMEF2^ with substantially smaller puncta compared to HDAC4^WT^ (Fig. 1D, arrows), thus overestimating the total amount of nuclear HDAC4 in these two mutants.

**Fig. 2:**
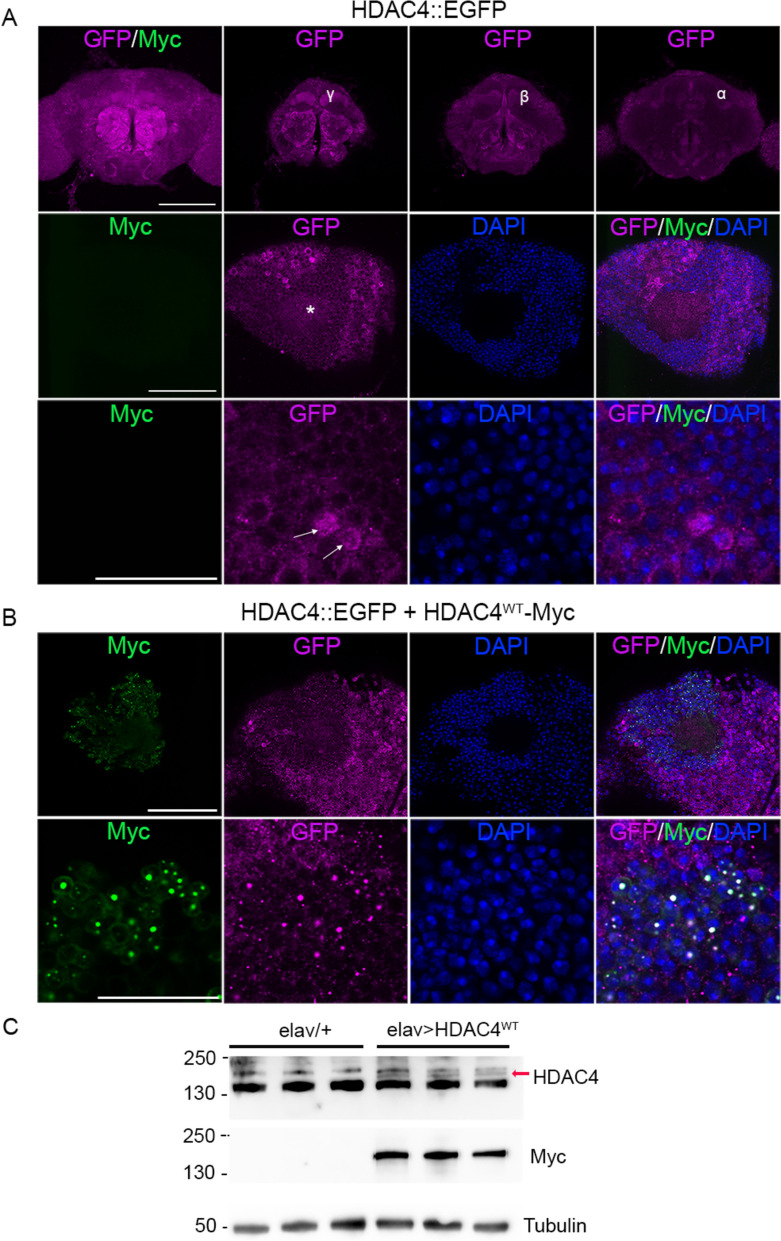
HDAC4 is endogenously expressed in the mushroom body and has similar distribution to the HDAC4^WT^ transgene. **A** Immunohistochemistry with anti-GFP on HDAC4::EGFP brains. Top row. The left-most image is a *Z*-stack maximum projection through the anterior of the brain showing the pattern of endogenous HDAC4 expression. The images to the right are 1-μm sections through the α, β and γ lobes of the mushroom body showing that HDAC4 is present in each of the lobes. Scale bar = 100 μm. Middle row. 0.5-μm optical sections through the Kenyon cell layer showing the distribution of HDAC4 in the calyx (asterisk) and cell bodies. Scale bar = 50 μm. Bottom row. Magnification of the images directly above, showing the cytoplasmic haloes around the nuclei (counterstained with DAPI), and punctate foci within some nuclei (arrows). Scale bar = 20 μm. **B** Immunohistochemistry with anti-GFP and anti-Myc on whole mount HDAC4::EGFP brains expressing HDAC4^WT^-Myc driven by *OK107-GAL4*. Note that constitutive *OK107* drives much stronger expression than maximal induction with GAL80ts; therefore, the aggregates are larger than in Fig. 1D. HDAC4^WT^-Myc is visible in in cytoplasmic haloes and in aggregates in a subset of nuclei. Within these nuclei, endogenous HDAC4 is also pulled into the aggregates. **C** Western blot of endogenous HDAC4 in the absence and presence of HDAC4^WT^ transgene expression. Anti-HDAC4 detects products ~ 135 kDa consistent with the estimated size of HDAC4 isoforms. When expressed with *elav-GAL4*, HDAC4^WT^ was detected at slightly larger molecular weight (red arrow) due to the 6xMyc tag, and the identity of HDAC4^WT^ was confirmed via detection of the Myc tag

We also compared the pattern of HDAC4^WT^ transgene expression to that of endogenous HDAC4 using HDAC4::EGFP, a protein trap line in which endogenous *HDAC4* carries an insertion of *EGFP* flanked by splice sites in the second intron, resulting in an internal fusion of EGFP within the translated protein [61]. A similar pattern in the mushroom body was observed, with localization to the lobes, calyx and cell bodies of Kenyon cells, with a subset of nuclei containing HDAC4 aggregated into puncta (Fig. 2A). When HDAC4^WT^ was expressed, endogenous HDAC4 colocalized in aggregates (Fig. 2B), which is consistent with HDAC4 homotetramerizing through its N-terminal α helix [52]. We confirmed that expression of HDAC4^WT^ resulted in only a modest increase in total HDAC4 (Fig. 2C) and thus was not a large increase over physiological levels.

In summary, HDAC4^WT^ and mutant transgenes were expressed at similar levels and displayed the expected distributions, with HDAC4^WT^ and HDAC4^Y1142H^ showing similar pattern to endogenous HDAC4 in the mushroom body. Of the subcellular distribution mutants, HDAC4^3SA^ was restricted to the nucleus where it formed large punctate aggregates, whereas HDAC4^ΔNLS^ and HDAC4^ΔMEF2^ resided largely outside the nucleus. Interestingly HDAC4^ΔANK^ displayed a more nuclear distribution than HDAC4^WT^. This panel of mutants therefore comprises valuable tool for molecular dissection of HDAC4, which we will characterize using models of mushroom body and eye development.

### Nuclear accumulation of HDAC4 impairs mushroom body development and impairment is exacerbated by loss of the ankyrin-repeat binding motif

**Fig. 3:**
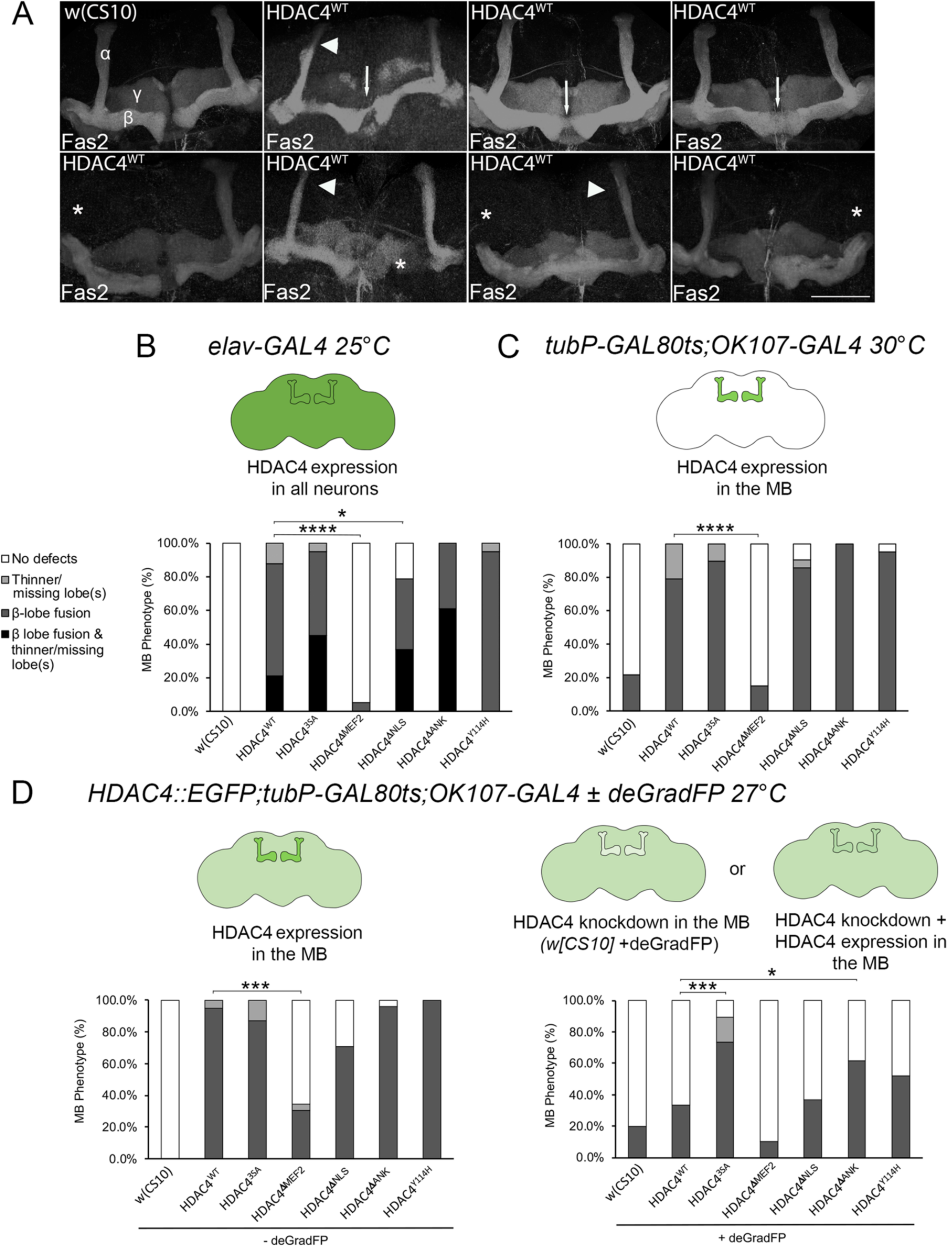
HDAC4^WT^ and mutants disrupt mushroom body development. **A** Immunohistochemistry with anti-Fas2 on whole mount brains expressing HDAC4^WT^ with *elav-GAL4*. Brains displaying representative phenotypes are shown. Scale bar = 75 μm. α, β and γ lobes of the mushroom body are labeled in white in a control brain in which *elav-GAL4* was crossed to *w(CS10)* (top left image). Thin lobes are indicated with arrowheads, β lobe fusion is indicated with arrows and missing lobes are indicated with asterisks. **B**–**D** Quantification of mushroom body phenotypes. Key shown in B applies to all four graphs. The percentage of brains displaying each phenotype is shown. **B** Quantification of phenotypes from brains expressing each of the mutants. To assist interpretation of the results, the cartoon above the graph indicates the relative approximate level of expression of the HDAC4^WT^ transgene by the intensity of the green shading. The proportion of brains displaying defects was significantly different between HDAC4^WT^ and HDAC4^ΔMEF2^, ****p < 0.00001, and HDAC4^WT^ and HDAC4^ΔNLS^, *p < 0.05, Fishers exact test. MB = mushroom body. **C** *tubP-GAL80ts; OK107-GAL4* females were crossed to males carrying each *UAS-HDAC4* transgene and to the *w(CS10)* control and raised at 30 °C for expression in the mushroom body of progeny throughout development. The proportion of brains displaying defects was significantly different between HDAC4^WT^ and HDAC4^ΔMEF2^, ****p < 0.0001, Fishers exact test. **D** Flies carrying *HDAC4::EGFP; tubP-GAL80ts; OK107-GAL4* and each *UAS-HDAC4* transgene or the *w(CS10)* control were raised at 27 °C for expression in the mushroom body throughout development in a WT (left graph) or HDAC4-depleted (right graph, + *UAS-deGradFP*) background. Light green in the cartoon indicates HDAC4::EGFP expression throughout the brain. Left graph. In the absence of deGradFP, there was no difference in the percentage of brains displaying defects between HDAC4^WT^ and any of the mutants except for HDAC4^ΔMEF2^, ***p < 0.001, Fisher’s exact test. Right graph. When endogenous HDAC4 was depleted with deGradFP and replaced with HDAC4^WT^ or mutants, there was no significant difference between HDAC4 knockdown + HDAC4^WT^, and HDAC4 knockdown alone (w[CS10] + deGradFP), indicating the overall level of HDAC4 expression was close to wild-type. However, in the deGradFP background, expression of HDAC4^3SA^ (***p < 0.001) and HDAC4^ΔANK^ (*p < 0.05) resulted in a significantly increased proportion of brains displaying defects compared to HDAC4^WT^, Fisher’s exact test

To determine the impact of expression of each of the mutants on axon morphogenesis, they were expressed with *elav-GAL4*, which is active during the larval and pupal stages when the mushroom body lobes develop [62]. Mushroom body integrity was subsequently assessed in adult brains via staining with anti-Fas2, which labels the α, β and γ lobes (Fig. 3A). Consistent with previous observations [41], *elav-GAL4*-driven expression of HDAC4^WT^ disrupts axon morphogenesis, which was predominantly observed as impaired elongation of α and β lobes as well as β lobe fusion, which is a result of β lobe axons crossing the midline to appear fused. Examples of these phenotypes are shown in Fig. 3A. When raised at 25 °C, all HDAC4^WT^ brains displayed abnormalities, with the majority displaying fusion of β lobes (Fig. 3B, Table 1). Nuclear accumulation of HDAC4 also resulted in a severe phenotype, with 95% of HDAC4^3SA^ brains displaying fused β lobes, and 45% of these brains also had thinner or absent α lobes. In contrast, 95% of HDAC4^ΔMEF2^ brains appeared normal. Interestingly, although predominantly cytoplasmic like HDAC4^ΔMEF2^, HDAC4^ΔNLS^ induced defects in 79% of brains. Since HDAC4^ΔNLS^ displayed a very similar distribution to HDAC4^ΔMEF2^, these data suggest that nuclear HDAC4 is not solely responsible for these deficits but that increased cytoplasmic HDAC4 also has the capacity to disrupt axon morphogenesis if the MEF2 binding region remains intact. All HDAC4^Y1142H^ and HDAC4^ΔANK^ brains were also abnormal, indicating that neither an intact catalytic site nor the ankyrin repeat motif are essential for the HDAC4 overexpression-induced impairment of axon morphogenesis.

**Table 1:** Frequency of mushroom body defects resulting from pan-neuronal expression of HDAC4^WT^ and mutants

Genotype	*n*	Total β lobe fusion (%)	β lobe fusion and thin or absent lobe(s) (%)	Severe β lobe fusion (%)	Moderate β lobe fusion (%)	Minor β lobe fusion (%)	Thin or absent lobe(s) (%)	No defects (%)
*elav/* +	19	0	0	0	0	0	0	100
*elav/* + *;UAS-HDAC4*^*WT*^*/* +	33	88	21	67	0	0	12	0
*elav/* + *;UAS-HDAC4*^*3SA*^*/* +	20	95	45	50	0	0	5	0
*elav/* + *;UAS-HDAC4*^*ΔMEF2*^*/* +	19	5	0	0	0	5	0	95
*elav/* + *;UAS-HDAC4*^*ΔNLS*^*/* +	19	79	37	26	11	5	0	21
*elav/* + *;UAS-HDAC4*^*ΔANK*^*/* +	18	100	61	33	6	0	0	0
*elav/* + *;UAS-HDAC4*^*Y1142H*^*/* +	20	95	0	65	30	0	5	0

The percentage of brains displaying a single phenotype of severe, moderate or minor β lobe fusion, or thin/absent lobe(s) is shown. As a measure of severity of the phenotype, the percentage of brains displaying both β lobe fusion and thin or absent lobes is also shown. The total percentage of brains displaying β lobe fusion is also calculated by combining minor, moderate and severe β lobe fusion, and those brains with both β lobe fusion and thin or absent lobes. *elav* = *elav-GAL4*

As these phenotypes are a result of increased ectopic expression, we also sought to express HDAC4^WT^ and mutants in presence of reduced endogenous HDAC4 via knockdown. This was intended to achieve an overall level of expression of HDAC4 that was close to that in wild-type brains. Expression was driven with *OK107-GAL4* so that HDAC4 knockdown was largely restricted to the mushroom body. Simultaneously, GAL80ts was also co-expressed. GAL80ts facilitates a linear increase in GAL4 activity on increase in temperature between 18 and 30 °C to allow fine tuning of the level of transgene expression [45, 60]. We confirmed that a very similar pattern of defects to those resulting from *elav-GAL4*-driven expression was observed when *OK107-*driven transgene expression was fully induced at 30 °C throughout development (Fig. 3C, Additional File 2, Table S1). Knockdown of endogenous HDAC4 was induced via the deGradFP genetic tool [63], which involves expression of a fusion protein containing the F-box domain from the N-terminus of the F-box protein Slmb (Nslmb) to a single domain antibody fragment that binds GFP (VhhGFP4). When Nslmb-vhhGFP4 binds GFP, it targets it for proteasomal degradation via complex formation with E3 ligase and ubiquitin-conjugating E2 enzymes. This method can therefore be used to reduce protein levels following expression of any gene tagged with GFP; in this case, we utilized the *HDAC4::EGFP* line in which endogenous HDAC4 is tagged with GFP. Knockdown of HDAC4 via deGradFP resulted in a mild impairment to mushroom body development (Additional File 2, Fig S2, Table S2A). This was confirmed with a second deGradFP line and an independent HDAC4::EYFP protein trap [64] (Additional File 2, Table S3). Efficient knockdown and HDAC4 transgene expression was confirmed by immunohistochemistry (Additional File 2, Fig S3).

We next determined the optimal level of expression at which endogenous HDAC4 was knocked down and replaced with the appropriate level of expression of HDAC4^WT^, so as to not induce a significant overexpression phenotype, and that the overall level of expression was close to that of endogenous HDAC4. Details of the validation are described in Additional File 2. At the optimal temperature of 27 °C, knockdown of HDAC4 induced a phenotype that was reduced by replacement with HDAC4^WT^ (Additional File 2, Figure S2, Table S2B). Expression of the HDAC4 mutants at 27 °C in resulted in the same pattern of defects as seen at 30 °C (Fig. 3D, left graph, Table 2), whereas in the reduced endogenous HDAC4 background, only HDAC4^3SA^ and HDAC4^ΔANK^ induced a significant increase in mushroom body defects compared to HDAC4^WT^ (Fig. 3D right graph, Table 3). A shift in the distribution of HDAC4 to the nucleus was disruptive to development, with 26% of HDAC4^3SA^ brains displaying severe β lobe fusion as compared to 7% of HDAC4^WT^. Similarly, there was also a significant increase in β lobe fusion in brains expressing HDAC4^ΔANK^, which is more nuclear in distribution than HDAC4^WT^ (Fig. 1G). The presence of an intact ankyrin repeat protein binding motif therefore appears to restrain the ability of HDAC4 to disrupt the processes required for normal axon morphogenesis by reducing nuclear accumulation.

**Table 2:** Frequency of mushroom body defects resulting from expression of HDAC4^WT^ or mutants in the mushroom body at 27 °C

Genotype	*n*	Total β lobe fusion	Severe β lobe fusion (%)	Moderate β lobe fusion (%)	Minor β lobe fusion (%)	Thin or absent lobe(s) (%)	No defects (%)
*HDAC4::EGFP/Y ;GAL80ts/* + *;OK107/* +	30	0	0	0	0	0	100
*HDAC4::EGFP/Y ;GAL80ts/* + *;UAS-HDAC4*^*WT*^*/* + *;OK107/* +	19	95	84	0	11	5	0
*HDAC4::EGFP/Y ;GAL80ts/* + *;UAS-HDAC4*^*3SA*^*/* + *;OK107/* +	23	87	74	9	4	13	0
*HDAC4::EGFP/Y ;GAL80ts/* + *;UAS-HDAC4*^*ΔMEF2*^*/* + *;OK107/* +	23	30	4	0	26	4	65
*HDAC4::EGFP/Y ;GAL80ts/* + *;UAS-HDAC4*^*ΔNLS*^*/* + *;OK107/* +	24	71	17	29	25	0	29
*HDAC4::EGFP/Y ;GAL80ts/* + *;UAS-HDAC4*^*ΔANK*^*/* + *;OK107/* +	20	100	65	25	10	0	0
*HDAC4::EGFP/Y ;GAL80ts/* + *;UAS-HDAC4*^*Y1142H*^*/* + *;OK107/* +	24	96	79	8	8	0	4

The percentage of brains displaying each of the defects is shown. The percentage of brains displaying a single phenotype of severe, moderate or minor β lobe fusion, or thin/absent lobe(s) is shown. No brains displayed both β lobe fusion and thin or absent lobes. The total percentage of brains displaying β lobe fusion is also calculated by combining minor, moderate and severe β lobe fusion, and those brains with both β lobe fusion and thin or absent lobes

**Table 3:** Frequency of mushroom body defects resulting from expression of HDAC4^WT^ and mutants in the mushroom body in an HDAC4-depleted background at 27 °C

Genotype	*n*	Total β lobe fusion (%)	Severe β lobe fusion (%)	Moderate β lobe fusion (%)	Minor β lobe fusion (%)	Thin or absent lobe(s) (%)	No defects (%)
**Control** *HDAC4::EGFP/Y ;GAL80ts/* + *;OK107/* +	30	0	0	0	0	0	100
**HDAC4 KD** *HDAC4::EGFP/Y ;GAL80ts/* + *;UAS-deGradFP/* + *;OK107/* +	25	20	4	8	8	0	80
**HDAC4 KD + HDAC4**^**WT**^ *HDAC4::EGFP/Y ;GAL80ts/* + *;UAS deGradFP/UAS-HDAC4*^*WT*^*;OK107/* +	30	33	7	13	13	0	67
**HDAC4 KD + HDAC4**^**3SA**^ *HDAC4::EGFP/Y ;GAL80ts/* + *;UAS-deGradFP/UAS-HDAC4*^*3SA*^*;OK107/* +	19	74	26	42	5	16	11
**HDAC4 KD + HDAC4**^**ΔMEF2**^ *HDAC4::EGFP/Y ;GAL80ts/* + *;UAS deGradFP/UAS-HDAC4*^*ΔMEF2*^*;OK107/* +	20	10	5	0	5	0	90
**HDAC4 KD + HDAC4**^**ΔNLS**^ *HDAC4::EGFP/Y ;GAL80ts/* + *;UAS deGradFP/UAS-HDAC4*^*ΔNLS*^*;OK107/* +	41	37	15	0	22	0	63
**HDAC4 KD + HDAC4**^**ΔANK**^ *HDAC4::EGFP/Y ;GAL80ts/* + *;UAS deGradFP/UAS-HDAC4*^*ΔANK*^*;OK107/* +	21	62	19	19	24	0	38
**HDAC4 KD + HDAC4**^**Y1142H**^ *HDAC4::EGFP/Y ;GAL80ts/* + *;UAS deGradFP/UAS-HDAC4*^*Y1142H*^*;OK107/* +	23	51	4	4	43	0	48

The percentage of brains displaying a single phenotype of severe, moderate or minor β lobe fusion, or thin/absent lobe(s) is shown. Flies were raised at 27 °C. No brains displayed both β lobe fusion and thin or absent lobes. The total percentage of brains displaying β lobe fusion is also calculated by combining minor, moderate and severe β lobe fusion, and those brains with both β lobe fusion and thin or absent lobes. KD, knockdown; *OK107* = *OK107-GAL4*

In summary, manifestation of the defects in mushroom body development resulting from nuclear accumulation of HDAC4 was dependent on the presence of the MEF2 binding region, but independent of the catalytic site, and exacerbated when the ankyrin repeat binding motif was mutated.

### CG5846, the Drosophila homologue of RFXANK, colocalizes with HDAC4 but does not regulate its distribution in the brain

**Fig. 4:**
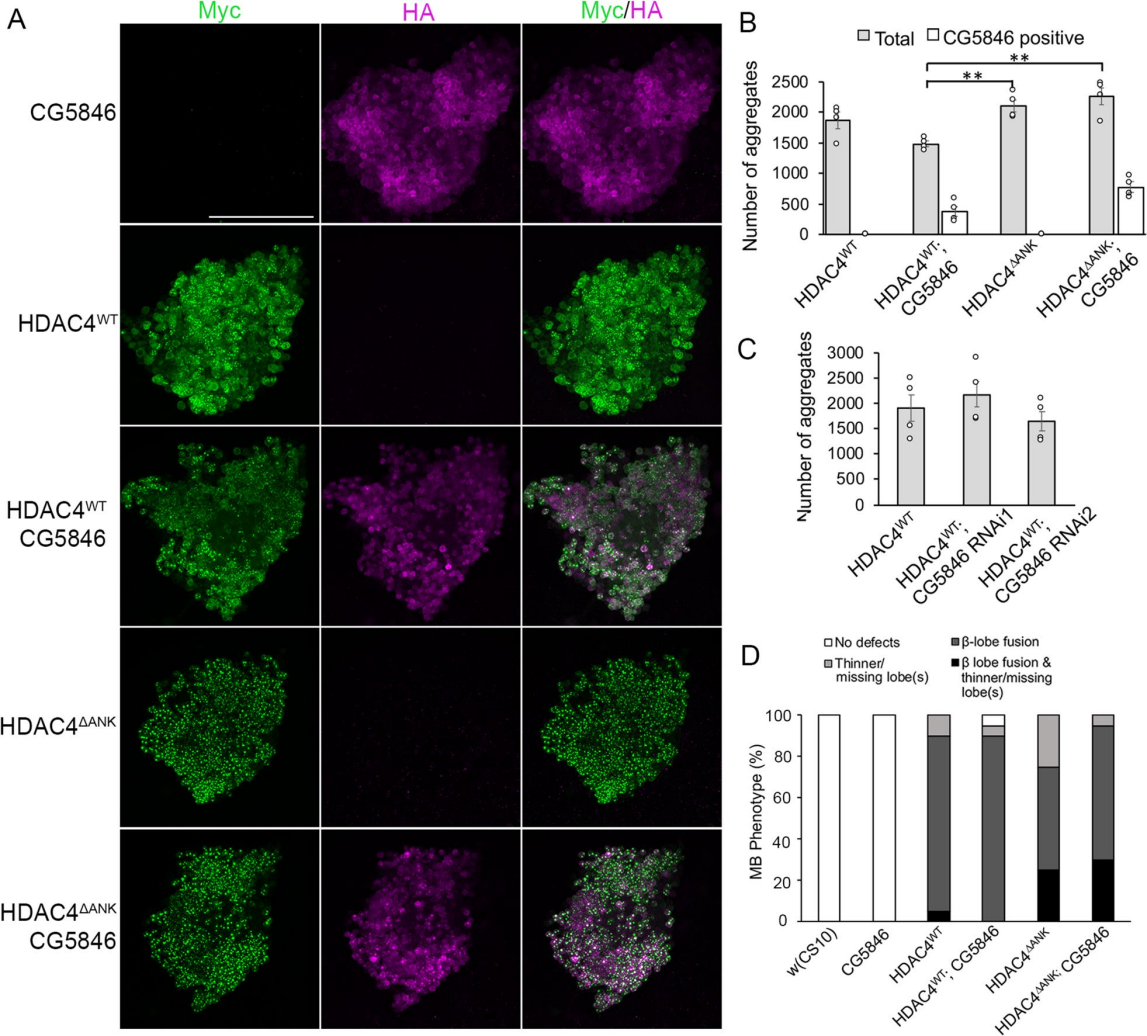
CG5846 is sequestered into HDAC4 aggregates but its expression does not alter the number of aggregates nor mushroom body phenotypes. **A** Immunohistochemistry on brains expressing *OK107-GAL4* driven Myc-tagged HDAC4^WT^ or HDAC4^ΔANK^ (green) in the presence and absence of HA-tagged CG5846 (magenta) in the mushroom body. Flies were raised at 23 °C. Representative maximum projections of 0.5-μm sections through the Kenyon cell bodies are shown. Scale bar = 50 μm. **B** Quantification of nuclear aggregates. Aggregates were counted through the maximum projection across the entire Kenyon cell layer, *n* = 4 brains/genotype. The number of aggregates was significantly increased in the Kenyon cell nuclei of HDAC4^ΔANK^ and HDAC4^ΔANK^;CG5846 brains in comparison to HDAC4^WT^;CG5846 (ANOVA, F_(3,12)_ = 9.03, *p* = 0.0021, Tukey’s post hoc test, **p < 0.01), but there was no significant difference between HDAC4^WT^ and HDAC4^WT^;CG5846, nor HDAC4^ΔANK^ and HDAC4^ΔANK^;CG5846. **C** Nuclear aggregates were quantified as in **B**. There was no significant change in aggregate number when CG5846 was knocked down (ANOVA, F_(2,9)_ = 0.98, *p* = 0.412). **D** Quantification of mushroom body phenotypes. The percentage of brains displaying each phenotype is shown. There was no significant difference in the proportion of brains displaying defects between those expressing HDAC4^WT^, HDAC4^WT^;CG5846, HDAC4^ΔANK^ and HDAC4^ΔANK^;CG5846 with the *elav-GAL4* driver

Given the increase in nuclear abundance of HDAC4 upon mutation of the ankyrin repeat binding motif, we hypothesized that protein binding at this site either tethers HDAC4 in the cytoplasm or aids in nuclear export. To that end, we investigated candidates that may interact with HDAC4 through this motif. We recently determined that HDAC4 and Ank2 do not physically interact in *Drosophila* [65]; therefore, our focus shifted to CG5846, a protein of close homology to RFXANK and ANKRA2, both of which interact with human HDAC4 through the PSLPNI motif [54–56]. When expressed in Kenyon cells with *OK107-GAL4*, CG5846 localized almost exclusively to nuclei, and appeared to concentrate around the nuclear periphery (Fig. 4A). Co-expression of HDAC4^WT^ altered the distribution of CG5846 such that it was sequestered into ~ 25% of the HDAC4 nuclear aggregates (Fig. 4A, B). The total number of HDAC4 aggregates was not significantly changed, and contrary to our hypothesis, many of the individual aggregates also appeared larger, indicating that CG5846 is not reducing nuclear abundance of HDAC4. The increased size of aggregates may account for the small (and insignificant) reduction in the total amount of aggregates in HDAC4^WT^ vs HDAC4^WT^;CG5846 brains. If the HDAC4-CG5846 interaction was via the PSLPNI motif, then the interaction would be lost in HDAC4^ΔANK^;CG5846 brains. However, while the overall total number of nuclear aggregates was higher in HDAC4^ΔANK^ brains, as seen previously (Fig. 1G), there was a similar percentage of aggregates containing CG5846 in HDAC4^WT^ and HDAC4^ΔANK^ brains. Therefore while we demonstrate proof of principle that CG5846 and HDAC4 colocalize in aggregates, this is not dependent on the presence of the PSLPNI motif. There was also no change in the number of nuclear aggregates when CG5846 was reduced in expression via RNAi in Kenyon cells (Fig. 4C), thus it does not appear to regulate HDAC4 subcellular distribution.

Analysis of mushroom body defects revealed no significant change when CG5846 was co-expressed with HDAC4^WT^ or HDAC4^ΔANK^ (Fig. 4D, Table 4), indicating that an interaction between HDAC4 and CG5846 is unlikely to be important for the HDAC4-induced defects in mushroom body development, thus further investigation of protein interactions at the PSLPNI motif and their impact on HDAC4 function are warranted.

**Table 4:** Co-expression of CG5846 does not alter the mushroom body defects induced by expression of HDAC4^WT^ or HDAC4^ΔANK^

Genotype	*n*	Total β lobe fusion (%)	β lobe fusion and thin or absent lobe(s) (%)	Severe β lobe fusion (%)	Moderate β lobe fusion (%)	Minor β lobe fusion (%)	Thin or absent lobe(s) (%)	No defects (%)
*elav/* +	20	0	0	0	0	0	0	100
*elav/* + *;UAS-CG5846/* +	20	0	0	0	0	0	0	100
*elav/* + *;UAS-HDAC4*^*WT*^*/* +	20	90	5	50	15	20	10	0
*elav/* + *;UAS-CG5846/* + *;UAS-HDAC4*^*WT*^	20	90	0	75	10	5	5	5
*elav/* + *;UAS-HDAC4*^*ΔANK*^*/* +	20	75	25	35	10	5	25	0
*elav/* + *;UAS-CG5846/* + *;UAS-HDAC4*^*ΔANK*^	20	95	30	55	10	0	5	0

The percentage of brains displaying a single phenotype of severe, moderate or minor β lobe fusion, or thin/absent lobe(s) is shown. Flies were raised at 23 °C. As a measure of severity of the phenotype, the percentage of brains displaying both β lobe fusion and thin or absent lobes is also shown. The total percentage of brains diplaying β lobe fusion is also calculated by combining minor, moderate and severe β lobe fusion, and those brains with both β lobe fusion and thin or absent lobes. *elav* = *elav-GAL4*

### HDAC4 acts through MEF2 to impair mushroom body development

**Fig. 5:**
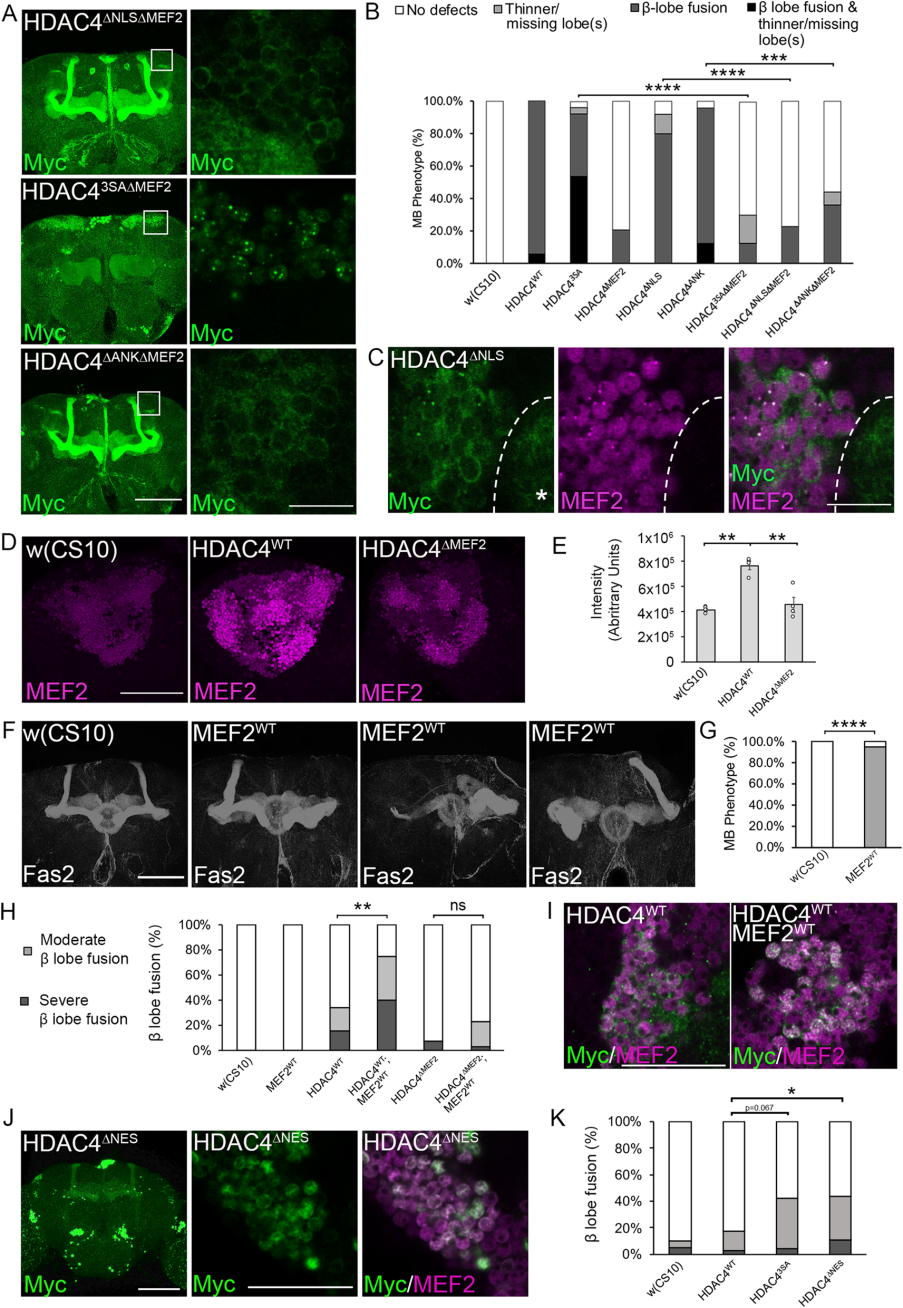
The MEF2 binding site is required for HDAC4-induced defects. **A** Myc immunohistochemistry on brains expressing the indicated Myc-tagged HDAC4 mutants in the mushroom body. Left panel: frontal projection through the mushroom body. Scale bar = 100 μm. Right panel: optical Sect. (0.5 μm) through the Kenyon cell bodies. Representative images are shown, scale bar = 10 μm. **B** Quantification of mushroom body phenotypes following expression of HDAC4^WT^ and mutants with *elav-GAL4*. The percentage of brains displaying each phenotype is shown. The proportion of brains displaying defects was significantly different between HDAC4^3SA^ and HDAC4^3SAΔMEF2^ (****p < 0.0001); between HDAC4^ΔNLS^ and HDAC4^ΔNLSΔMEF2^ (****p < 0.0001) and between HDAC4^ΔANK^ and HDAC4^ΔANKΔMEF2^ (***p < 0.001), Fisher’s exact test. **C** Myc (green) and MEF2 (magenta) immunohistochemistry on brains expressing HDAC4^ΔNLS^ as described in **A**. Optical Sects. (0.5 μm) through the Kenyon cell bodies are shown. HDAC4^ΔNLS^ is present in cytoplasmic haloes and in the calyx but MEF2 is only present in nuclei and does not overlap with HDAC4^ΔNLS^ outside the nucleus. Scale bar = 10 μm. **D** *OK107-GAL4*-driven expression of HDAC4^WT^ and HDAC4^ΔMEF2^ was induced in the adult brain with GAL80ts by raising the temperature from 18 to 30 °C at 3 days post-eclosion for 72 h, followed by anti-MEF2 immunohistochemistry. Maximum projections through the Kenyon cell layer are shown. Scale bar = 50 μm. **E** Quantification of MEF2 intensity, *n* = 4 brains/genotype (ANOVA, F_(2,9)_ = 22.61, *p* = 0.0003, Tukey’s post hoc test, **p < 0.01). **F** HA-tagged wild-type MEF2 (MEF2^WT^) was expressed with *elav-GAL4*. Frontal projections through the mushroom body of brains expressing MEF2^WT^ stained with Fas2 are shown in comparison to the control (*elav-GAL4* crossed to *w[CS10])*. Overexpression of MEF2 results in defects in axon elongation and guidance. Scale bar = 50 μm. **G** Quantification of mushroom body phenotypes following Fas2 staining as shown in **F** and Table 4. The key is the same as shown in **B**, ****p < 0.0001, Fisher’s exact test. **H**–**J** The indicated HDAC4 and MEF2 transgenes were expressed to approximately half maximal GAL80ts-induced levels by raising flies at 25 °C. **H** Co-expression of HDAC4^WT^ and MEF2^WT^ resulted in a significantly higher proportion of brains with β lobe fusion than HDAC4^WT^ alone, **p < 0.01, Fisher’s exact test. There was no significant difference between HDAC4^ΔMEF2^ and HDAC4^ΔMEF2^ co-expressed with MEF2^WT^, *p* = 0.1468, Fisher’s exact test. **I** When co-expressed with MEF2^WT^, HDAC4^WT^ was more nuclear as observed by the increased colocalization with MEF2 (white), and the reduced cytoplasmic halos (green) that were observed when HDAC4^WT^ was individually expressed. Scale bar = 20 μm. **J** Left image: A frontal projection through the mushroom body lobes shows that HDAC4^ΔNES^ is almost completely restricted to nuclei. The intensity of the image has been increased to show the outline of the brain and location of the mushroom body lobes, which were not visible at the same confocal gain settings used to visualize HDAC4^WT^ in the lobes. Scale bar = 100 μm. Right images: HDAC4^ΔNES^ colocalizes in with MEF2 in Kenyon cell nuclei. Scale bar = 20 μm. **K** Expression of HDAC4^ΔNES^ resulted in a significantly higher proportion of brains with β lobe fusion than HDAC4^WT^, Fisher’s exact test, *p < 0.05

The discovery that expression of the two cytoplasmically localized mutants HDAC4^ΔNLS^ and HDAC4^ΔMEF2^ resulted in significantly different phenotypes raised the question of whether the disruption to development caused by HDAC4^ΔNLS^ is also dependent on the presence of the MEF2 binding site. This observation is intriguing, given that MEF2 is a transcription factor that localizes to nuclei of Kenyon cells [40]. To address this, we generated the double mutant HDAC4^ΔNLSΔMEF2^, which also localized to the cytoplasm (Fig. 5A). The phenotype resulting from expression of HDAC4^ΔNLSΔMEF2^ was also significantly reduced compared to HDAC4^ΔNLS^ (Fig. 5B, Table 5), confirming that the MEF2 binding region is required for HDAC4^ΔNLS^-induced disruption of axon development. To test whether the HDAC4^ΔNLS^ phenotype might result from sequestering of MEF2 in the cytoplasm and thus reducing its activity in the nucleus, the distribution of endogenous MEF2 was investigated but it was only detected in the nucleus with a lack of overlap between HDAC4^ΔNLS^ and MEF2 in the cytoplasm or calyx (Fig. 5C). It should be noted that while a very low level of HDAC4^ΔNLS^ was observed in the nucleus, it was still largely cytoplasmic and the aggregates are few and pinpoint as compared to HDAC4^WT^ (compare Fig. 1D HDAC4^WT^ with HDAC4^ΔNLS^). Nuclear entry is inefficient without an NLS [4, 20], and the very low levels of HDAC4^ΔNLS^ in the nucleus are likely due to tetramerization of endogenous HDAC4 with HDAC4^ΔNLS^.

**Table 5:** Impact of mutation of the MEF2 binding region on the frequency of mushroom body defects resulting from pan-neuronal expression of HDAC4^WT^ and mutants

Genotype	*n*	Total β lobe fusion (%)	β lobe fusion and thin or absent lobe(s) (%)	Severe β lobe fusion (%)	Moderate β lobe fusion (%)	Minor β lobe fusion (%)	Thin or absent lobe(s) (%)	No defects (%)
*elav/* +	23	0	0	0	0	0	0	100
*elav/* + *;UAS-HDAC4*^*WT*^*/* +	18	100	6	92	0	0	0	0
*elav/* + *;UAS-HDAC4*^*3SA*^*/* +	26	92	54	35	4	0	4	4
*elav/* + *;UAS-HDAC4*^*ΔMEF2*^*/* +	24	21	0	0	0	21	0	79
*elav/* + *;UAS-HDAC4*^*ΔNLS*^*/* +	25	80	0	4	36	40	12	8
*elav/* + *;UAS-HDAC4*^*ΔANK*^*/* +	24	96	13	79	4	0	0	4
*elav/* + *;UAS-HDAC4*^*3SAΔMEF2*^*/* +	23	12	0	0	4	9	17	70
*elav/* + *;UAS-HDAC4*^*ΔNLSΔMEF2*^*/* +	22	23	0	0	5	18	0	77
*elav/* + *;UAS-HDAC4*^*ΔANKΔMEF*^*/* +	25	36	0	0	0	36	8	56
*elav/* + *;UAS-MEF2*^*WT*^*/* +	18	0	0	0	0	0	90	10

The percentage of brains displaying a single phenotype of severe, moderate or minor β lobe fusion, or thin/absent lobe(s) is shown. Flies were raised at 25 °C. As a measure of severity of the phenotype, the percentage of brains displaying both β lobe fusion and thin or absent lobes is also shown. The total percentage of brains diplaying β lobe fusion is also calculated by combining minor, moderate and severe β lobe fusion, and those brains with both β lobe fusion and thin or absent lobes. *elav* = *elav-GAL4*

We also introduced the MEF2 binding region mutations into HDAC4^ΔANK^. The resulting HDAC4^ΔANKΔMEF2^ double mutant was only detected in the cytoplasm (Fig. 5A) due to the lack of MEF2-dependent import, and consequently, its ability to impair mushroom body development was reduced to a similar level to HDAC4^ΔMEF2^ (Fig. 5B, Table 5).

We recently showed that the HDAC4^3SA^ phenotype in the mushroom body was significantly reduced on mutation of the MEF2 binding region (HDAC4^3SAΔMEF2^) [41]. Despite this, HDAC4^3SAΔMEF2^ still accumulates largely in the nucleus (Fig. 5A). While the NLS is inefficient and MEF2 binding is required for efficient nuclear entry [4, 20], HDAC4 can enter at a low level [4], and the 3SA mutations prevent phosphorylation-induced nuclear exit [4, 13–18], thus resulting in nuclear accumulation over time. A small amount of HDAC4^3SAΔMEF2^ in the mushroom body lobes is observed, demonstrating the slow nuclear accumulation over time compared to HDAC4^3SA^, which is absent from the lobes (Fig. 1D). We quantified the number of HDAC4^3SAΔMEF2^ nuclear aggregates and found that compared to HDAC4^3SA^ there were a similar number of aggregates that did not contain MEF2. However, the number of MEF2-containing aggregates was approximately 50% lower in HDAC4^3SAΔMEF2^ nuclei, and the MEF2 intensity was also reduced (Additional File 1). This indicates that the reduced mushroom body defects resulting from expression of HDAC4^3SAΔMEF2^ are not simply due to reduced nuclear HDAC4, but that for HDAC4 to exert its effects, MEF2 binding in the nucleus is required. The mechanism through which HDAC4 and MEF2 act to impair development is not clear. If the deleterious effects on development were a result of the traditionally understood interaction between the two proteins in which HDAC4 binds to and represses MEF2, then we would expect that knockdown of MEF2 in the presence of HDAC4 would also impair development. However on the contrary, when MEF2 RNAi was co-expressed with HDAC4^3SA^, mushroom body development was normal in most brains [41], which shows that HDAC4 is dependent on the presence of MEF2 to disrupt development. Interestingly, while quantifying MEF2-containing aggregates, we observed that expression of HDAC4^WT^ but not HDAC4^ΔMEF2^ resulted in increased intensity of MEF2 in Kenyon cell nuclei (Fig. 5D, E) which suggests that HDAC4^WT^ acts to stabilize or upregulate endogenous MEF2. We therefore examined whether the impairments to development could be reproduced by increased expression of MEF2 and found that it resulted in severe defects in axon elongation and guidance in almost all brains (Fig. 5F, G, Table 5). It is clear that increased MEF2 is indeed detrimental; however while the phenotypes overlapped with those induced by increased expression of HDAC4, none of the MEF2^WT^ expressing brains displayed the predominant HDAC4 phenotype of β lobe fusion. Therefore phenotypic manifestation of β lobe fusion requires not just MEF2 but also an increased abundance of HDAC4 capable of binding MEF2.

To further examine this, we reasoned that co-expression of MEF2^WT^ with HDAC4^WT^ would result in increased nuclear accumulation of HDAC4 via MEF2-dependent entry and a subsequent increase in β lobe fusion. Since expression of HDAC4^WT^ alone already results in β lobe fusion in most brains (Table 1), we reduced its expression to approximately half maximum with GAL80ts. Accordingly, only 35% β lobe fusion was observed on individual expression of HDAC4^WT^, but when co-expressed with MEF2^WT^, this increased to 75% (Fig. 5H, Table 6), and correlated with an increase in nuclear HDAC4 (Fig. 5I). Notably, co-expression of MEF2^WT^ with HDAC4^ΔMEF2^ did not significantly increase the severity of β lobe fusion (Fig. 5H) demonstrating that the increased severity of HDAC4^WT^ is specifically via the MEF2 binding region.

**Table 6:** Increased nuclear accumulation of HDAC4 via co-expression of MEF2 results in increased β lobe fusion compared to HDAC4^WT^

Genotype	*n*	Total β lobe fusion (%)	Severe β lobe fusion (%)	Moderate β lobe fusion (%)	No defects (%)
*GAL80ts/* + *;OK107/* +	30	0	0	0	100
*GAL80ts/UAS-MEF2*^*WT*^*/* + *;OK107/* +	29	0	0	0	100
*GAL80ts/+ ;UAS-HDAC4*^*WT*^*;OK107/* +	26	35	15	19	65
*GAL80ts/UAS-MEF2*^*WT*^/ + ;*UAS-HDAC4*^*WT*^/ + ;*OK107/* +	20	75	40	35	25
*GAL80ts/+ ;UAS-HDAC4*^*ΔMEF2*^*/* + *;OK107/* +	28	7	7	0	93
*GAL80ts/UAS-MEF2*^*WT*^/ + ;*UAS-HDAC4*^*ΔMEF2*^/ + ;*OK107/* +	30	23	3	20	77

The percentage of brains displaying moderate or severe β lobe fusion is shown. Transgenes were expressed with *OK107-GAL4* in the presence of *tubP-GAL80ts*. Flies were raised at 25 °C to achieve approximately half the maximal induction of expression. The total percentage of brains diplaying β lobe fusion is also calculated by combining all columns containing β lobe fusion

Taken together, these data show that increased nuclear HDAC4 correlates with more severe defects in mushroom body development and that this is dependent on MEF2. These data correlating increased nuclear abundance of HDAC4 with more severe phenotypes also allay a potential concern that phenotypes resulting from expression of HDAC4^3SA^ could be due to other possible effects of the 3SA mutations rather than the nuclear accumulation itself. To that end, we mutated the nuclear export sequence of HDAC4 (HDAC4^ΔNES^), which also resulted in nuclear accumulation and depletion from the mushroom body lobes (Fig. 5J), and more severe β lobe fusion than HDAC4^WT^ (Fig. 5K, Table 7).

**Table 7:** Expression of HDAC4^ΔNES^ results in increased β lobe fusion compared to HDAC4^WT^

Genotype	*n*	Total β lobe fusion (%)	Severe β lobe fusion (%)	Moderate β lobe fusion (%)	No defects (%)
*GAL80ts/* + *;OK107/* +	20	10	5	5	90
*GAL80ts/+ ;UAS-HDAC4*^*WT*^*/+ ;OK107/* +	29	17	3	14	83
*GAL80ts/+ ;UAS-HDAC4*^*3SA*^*/+ ;OK107/* +	24	42	4	38	58
*GAL80ts/+ ;UAS-HDAC4*^*ΔNES*^*/+ ;OK107/* +	27	44	11	33	56

The percentage of brains displaying moderate or severe β lobe fusion is shown. Transgenes were expressed with *OK107-GAL4* in the presence of *tubP-GAL80ts*. Flies were raised at 25 °C to achieve approximately half the maximal induction of expression. The total percentage of brains diplaying β lobe fusion is also calculated by combining all columns containing β lobe fusion

During the course of these analyses, we noticed that expression of HDAC4^WT^ reduced the levels of the neuronal cell adhesion molecule Fas2 in the mushroom body (Additional File 3). While being used in this instance to label the mushroom body lobes, this phenotype piqued interest as Fas2 was identified as a MEF2 target via ChIP-chip on heads of flies expressing MEF2 [66]. Multiple differentially expressed splice variants of Fas2 exist, some of which contain a transmembrane domain and others that are GPI-anchored to the membrane, the latter of which are not detected by the 1D4 Fas2 antibody [67–69]. Interestingly, HDACs [70] including HDAC4 [71] have been implicated in regulation of alternative splicing. Further investigation revealed that in addition to HDAC4^WT^, HDAC4^3SA^ and HDAC4^ΔANK^, the three mutants that promoted the most severe mushroom body phenotypes also reduced the levels of Fas2, and in each case, this was reversed by mutation of the MEF2 binding region. We observed no change in Fas2 on overexpression or knockdown of MEF2 alone (Additional File 3). It is unclear whether HDAC4 regulates transcription, splicing, protein stability or is acting through another mechanism, but these data indicate that nuclear HDAC4 with an intact MEF2 binding region is required both to reduce Fas2 and to disrupt mushroom body development.

### HDAC4-induced defects in photoreceptor differentiation are dependent on an intact catalytic site and only minimally dependent on an intact MEF2 binding site

**Fig. 6:**
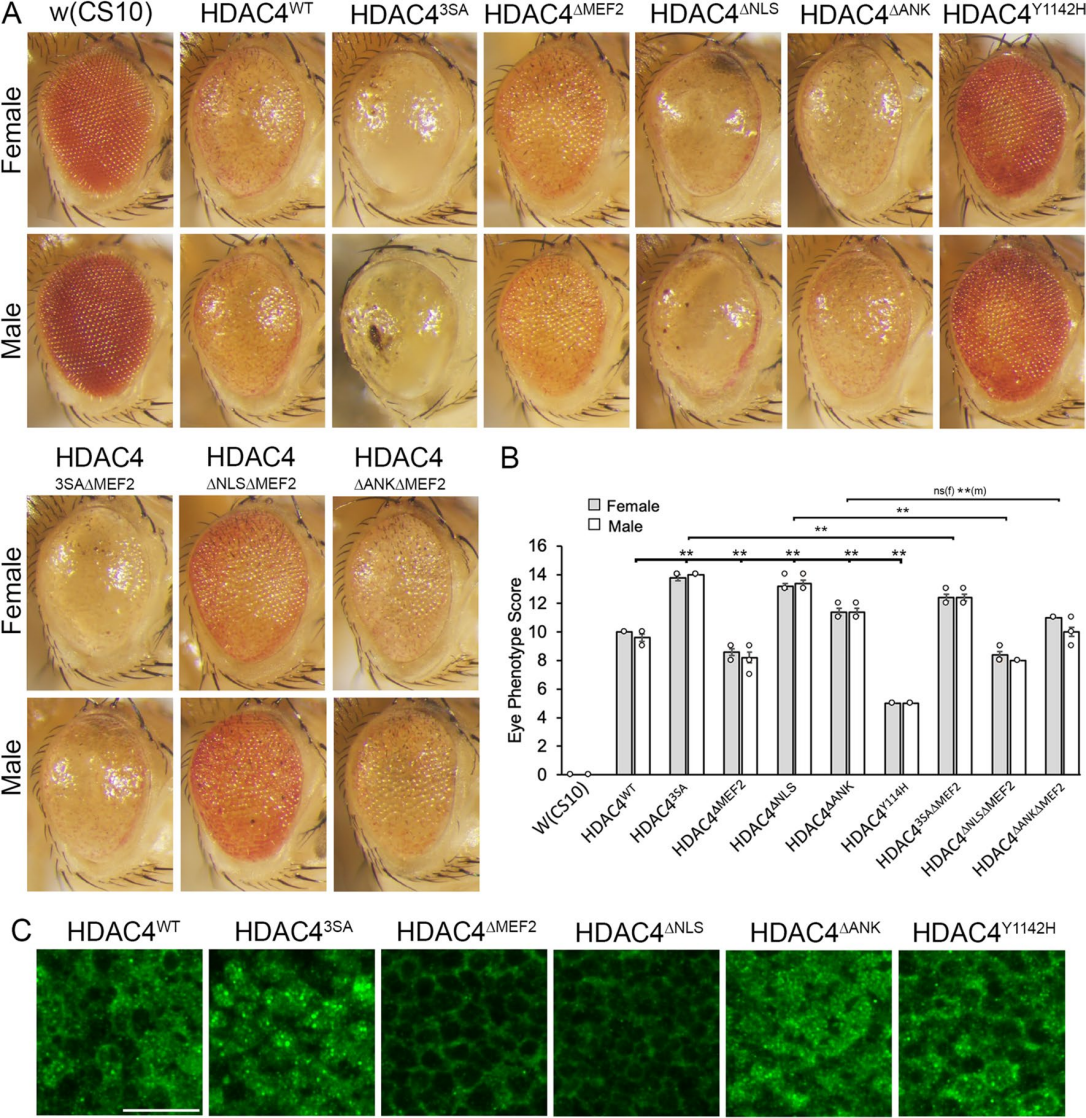
Expression of nuclear and cytoplasmic-restricted HDAC4 exacerbates disruption to eye development, which is reduced in the Y1142H mutant.** A** Stereomicrographs of *Drosophila* eyes. Genotypes were generated by crossing *GMR-GAL4/CyO;UAS-HDAC4* females to males carrying each *UAS-HDAC4* transgene and to the *w(CS10)* control and collecting non Cy progeny. Representative images of *n* = 5 males and *n* = 5 females per genotype are shown. × 110 magnification. **B** Quantification of the severity of eye phenotypes. In comparison to HDAC4^WT^, the severity of HDAC4^3SA^, HDAC4^ΔANK^, and HDAC4^ΔNLS^ phenotypes was significantly increased. In contrast, the HDAC4^ΔMEF2^ and HDAC4^Y1142H^ phenotypes were both significantly reduced in males and females (females: ANOVA, F(8,36) = 218.5, *p* = 1.11 × 10^−16^. Tukey’s post hoc test, **p < 0.01; males: ANOVA, F(8,36) = 157.58, *p* = 1.11 × 10^−16^. Tukey’s post hoc test, **p < 0.01). Error bars = mean ± SEM. **C** Anti-Myc immunohistochemistry on eye imaginal discs from wandering third instar larvae expressing HDAC4^WT^ and mutants with *GMR-GAL4*. Representative images from *n* = 15 discs per genotype are shown. Scale bar = 10 μm

In addition to the severe disruption to brain development, expression of HDAC4^WT^ also perturbs development of photoreceptors, which manifests as ommatidial fusion, disruption of bristle formation as well as loss of pigmentation. The phenotype is sensitive to dose, with two copies of HDAC4 resulting in a phenotype more severe than one copy [45]. The ability to readily assess the severity of these phenotypes in a semi-quantitative manner makes this an ideal model to determine whether the MEF2-dependent mechanism through which HDAC4 disrupts development can be generalized to other neuronal types. Flies were generated that harbored two copies of either HDAC4^WT^ or each mutant and one copy of *GMR-GAL4*. Similarly to the effect on the mushroom body development, the HDAC4^WT^ phenotype was exacerbated by nuclear-restriction of HDAC4 (Fig. 6A, B). Expression of HDAC4^ΔMEF2^ induced a phenotype that was reduced in comparison to HDAC4^WT^ but only mildly so; therefore, unlike in the mushroom body, the MEF2 binding region plays a minimal role in manifestation of the HDAC4^WT^-induced eye phenotype. Interestingly, expression of HDAC4^ΔNLS^ resulted in a phenotype more severe than HDAC4^WT^, indicating that cytoplasmic HDAC4 is neurotoxic in the eye and is more severe when the MEF2 binding region is intact, as the HDAC4^ΔNLSΔMEF2^ double mutant was comparable to HDAC4^ΔMEF2^. Expression of HDAC4^ΔANK^ also disrupted eye development more so than HDAC4^WT^; therefore, the intact ankyrin binding domain restrains the ability of HDAC4 to impair development in both the mushroom body and the eye. Interestingly, in contrast to that observed in the mushroom body, mutation of the active site in the HDAC4^Y1142H^ mutant reduced the severity of the phenotype considerably, demonstrating that an intact catalytic site plays an important role in HDAC4-induced developmental defects in the eye.

To allow analysis of the phenotypes with respect to subcellular distribution, we investigated the expression of the HDAC4 mutants in eye imaginal discs and found that their distribution largely correlated with that seen in the mushroom body (Fig. 6C). HDAC4^WT^ was detected in the cytoplasm of all cells and formed aggregates in a proportion of nuclei in the basal cells of the disc, as did HDAC4^Y1142H^ and HDAC4^ΔANK^, with HDAC4^ΔANK^ present in a larger proportion of nuclei than HDAC4^WT^. HDAC4^3SA^ distributed to nuclei, whereas HDAC4^ΔMEF2^ and HDAC4^ΔNLS^ were cytoplasmic.

The modest impact of mutation of the MEF2 binding region on HDAC4^3SA^ and HDAC4^ΔANK^-induced phenotypes (Fig. 6A, B) was in stark contrast to our observations in the mushroom body (Fig. 5B). This prompted us to examine the expression pattern of MEF2 in the developing eye, which has to our knowledge not been characterized. The ability of the antibody to detect MEF2 was confirmed by expression of a MEF2-HA transgene, which was detected with the MEF2 antibody in an identical pattern to that of the HA antibody in the mushroom body and eye imaginal disc; however, no expression of endogenous MEF2 was detected in eye discs (Additional File 4). We note that transcript expression of MEF2 has been detected via RNA-seq in the larval eye-antennal disc; however, this was at very low levels [72]. Furthermore, MEF2 clustered with transcription factors which localize anterior to the morphogenetic furrow and are decreased in expression levels at the onset of differentiation [72]. Given our HDAC4-overexpression-induced phenotypes were mediated via *GMR-GAL4*, which induces expression posterior to the morphogenetic furrow [73], it is unlikely that HDAC4 undergoes significant MEF2-dependent regulation in these cells. This therefore provides a molecular basis for the modest impact of deletion of the MEF2 binding site, and these data together indicate that mechanism of action of HDAC4 in the eye differs from that in the mushroom body and appears to be more dependent on an intact deacetylase domain.

## Discussion

Understanding the roles of nuclear and cytoplasmic HDAC4 and mechanisms regulating its nuclear entry and exit is an area of concerted effort due to the association of nuclear accumulation of HDAC4 with neurodevelopmental and neurodegenerative disease. Here we have generated a series of HDAC4 mutants to dissect the importance of specific features of the HDAC4 protein to its function in two models of neuronal development.

We found that in the mushroom body, forced sequestration of HDAC4 in the nucleus or the cytoplasm was detrimental to development. The actions of HDAC4 that resulted in impaired development were dependent on MEF2 binding, modulated by the PSLPNI motif, and largely independent of an intact catalytic site. This was not a consequence of overexpression, as expression at a physiological level to model shuttling defects via depletion of endogenous HDAC4 still impaired development when HDAC4 accumulated in the nucleus via either mutation of 14–3-3 binding sites (HDAC4^3SA^) to prevent nuclear exit, or mutation of the ankyrin repeat binding motif (HDAC4^ΔANK^).

When the MEF2 binding region within HDAC4 was mutated, the mushroom body defects were greatly reduced. HDAC4 is traditionally known as a transcriptional repressor of MEF2 whereby it binds MEF2 to prevent activation of MEF2 target genes, which would be consistent with mutation of the MEF2 binding site resulting in de-repression of MEF2. However, this appears not to be the case, as MEF2 knockdown would be expected to result in a further impairment, but we previously showed that co-expression of HDAC4^3SA^ and a MEF2 inverted repeat hairpin ameliorates the HDAC4^3SA^-induced phenotype in the mushroom body [41]. In fact, it appears that the opposite is the case, since expression of HDAC4^WT^ increased the amount of MEF2 in Kenyon cells, thus the mushroom body defects correlate with increased MEF2. The mechanism through which HDAC4 expression results in increased MEF2 requires further investigation; however, we surmise that it is stabilizing MEF2 rather than transcriptionally upregulating it, because we saw no increase in MEF2 mRNA when we measured transcriptional changes resulting from HDAC4 overexpression via RNA-seq [45]. HDAC4 does not appear to be disrupting mushroom body development simply by upregulating MEF2, since when we tested this by overexpressing MEF2, we did not see any instances of β lobe fusion, which was the predominant HDAC4-induced phenotype. It is possible that sequestration of MEF2 into aggregates results in recruitment of other proteins that together disrupt normal function. Fas2 has been implicated in axon morphogenesis in the mushroom body; defects including thin dorsal lobes and fused medial lobes have been observed in hypomorphic *Fas2* mutant larvae [74], although these defects do not persist into adulthood due to remodelling that occurs during pupation [75, 76]. Ectopic expression of Fas2 in α′/β′ neurons also results in lobe branching defects [76]. The normal appearance of the mushroom body in *Fas2* null mutants indicates that downregulation of Fas2 alone is not the underlying cause of the mushroom body defects that we observed, but it is likely not the only HDAC4-MEF2 target and perhaps Fas2 acts in concert with other factors that are also altered by increased nuclear HDAC4. Another possibility is a disruption to splicing; Fas2 splice variants differ in expression and function; only the neuronal transmembrane isoform of Fas2 can rescue splicing-related morphological defects in synapse development in the larval neuromuscular junction [77]. It will therefore be of interest to determine whether the presence of HDAC4-MEF2 aggregates alters Fas2 splicing, and if so, whether the expression of other splice variants not normally expressed in the mushroom body is detrimental. Further research such as targeted DamID in Kenyon cells will also be informative in identification of HDAC4 and MEF2 targets in chromatin and whether formation of aggregates alters association with chromatin.

Unlike in the mushroom body, the MEF2 binding region played only a minor role in disruption to ommatidial development as the phenotype resulting from expression of HDAC4^ΔMEF2^ was only slightly less severe than of HDAC4^WT^. However, expression of the HDAC4^ΔNLS^ mutant, in which HDAC4 is largely cytoplasmic but retains the MEF2 binding region, induced a severe phenotype in both the mushroom body and the eye. This is curious given that MEF2 is a nuclear transcription factor and also was not detectable in the developing eye. The domain surrounding the MEF2 binding region binds other factors, and HDAC4 has been identified to interact with several transcription factors that have homologues in *Drosophila* including Forkhead box class O (Foxo) [78], Activating transcription factor 4 (ATF4) [11], Serum response factor (SRF) [1], cAMP response element binding protein (CREB) [27], hypoxia-inducible factor-1a (HIF-1a) [79], C-terminal binding protein (CtBP) [80], and Runt-related transcription factor 2 (RUNX2) [3]. For the majority of these transcription factors, the specific binding domain has not yet been characterized, and their interactions with HDAC4 have not been investigated in *Drosophila* neurons. The binding domain of murine RUNX2 has been narrowed down within first 220 amino acids of HDAC4, which includes the MEF2 binding domain [3], and the SRF binding domain has also been localized to aa 1–289 of rodent HDAC4, with the highest affinity binding requiring the presence of amino acids 201–289, a region adjacent to the MEF2 binding domain [1]. It is therefore likely that other proteins interact at this site and that mutation of the MEF2 binding site alters the interaction of other factors at overlapping or nearby sites. Also of potential significance is PP2A, which dephosphorylates several serine residues to facilitate nuclear entry of HDAC4. Its binding site has been mapped to the same domain as the MEF2 binding region in human HDAC4 [19]. If PP2A is unable to bind HDAC4^ΔMEF2^ then this would reinforce its cytoplasmic localization and the change in its phosphorylation status could alter interactions with other HDAC4 binding partners.

Mutation of the catalytic tyrosine residue had little effect on mushroom body development but severely reduced the HDAC4-induced defects in the eye. It has been previously shown that nuclear sequestration of HDAC4 in the mouse forebrain induces defects in memory acquisition and retention, which occur in the absence of the catalytic domain [36]. The C-terminus also binds the HDAC3 corepressor complex [7, 81] and although it has little intrinsic deacetylase activity, it retains binding to ε-N-acetyl lysine peptides in vitro and thus likely recruits proteins via their acetyl lysine side chains. While substitution of tyrosine 1142 severely reduced the development defects in the eye, the eyes were clearly still abnormal in appearance; therefore, it is likely that that HDAC4 also acts through deacetylase-independent mechanisms, and/or potentially still confers some deacetylase activity through tetramerization with endogenous HDAC4 to deacetylate histone or non-histone targets in the nucleus.

There has been little investigation of proteins that interact with HDAC4 via the PSLPNI motif and the impact on HDAC4 function. Mammalian HDAC4 has been identified to interact with RFXANK [55, 56] and ANKRA2 [54–56] via direct binding to the PSLPNI motif [56], which is 100% conserved in *Drosophila*. We found that mutation of the PSLPNI motif to ASAANA resulted in more severe phenotypes in both brain and eye than HDAC4^WT^ and correlated with an increased number of aggregates in Kenyon cells. This suggests ankyrin repeat-containing protein(s) modulate nuclear entry or promote nuclear exit of HDAC4 such that when they cannot bind, there is a shift towards nuclear accumulation. Despite this, co-expression of CG5846, the *Drosophila* homologue of RFXANK, did not alter HDAC4 distribution nor the mushroom body phenotype. CG5846 also did not appear to interact differentially with HDAC4^ΔANK^ compared to HDAC4^WT^ suggesting it is not the protein responsible for changes in mushroom body or eye phenotypes resulting from HDAC4^ΔANK^ mutation. In addition to the PSLPNI motif providing a docking site for ankyrins, the serine residue within the motif can be phosphorylated to create an additional binding site for 14–3-3 proteins, which prevents binding of ANKRA2 or RFXANK, thereby acting as a switch to modulate protein–protein interactions [56], in addition to facilitating stronger nuclear export. The HDAC4^ΔANK^ mutant is unlikely to effectively bind 14–3-3 proteins as the residues surrounding the serine (which are mutated in HDAC4^ΔANK^) also form a network of hydrogen bonds that are required for 14–3-3 binding in vitro [56]; therefore, mutation of the motif to reduce 14–3-3 binding may also be a contributor to the increased nuclear presence of HDAC4. Given that mutations to other 14–3-3 binding sites in HDAC4 result in neurodevelopmental deficits presumably due to a shift in nucleocytoplasmic distribution of HDAC4 [37], further investigation into the regulation of the PSLPNI motif phosphorylation and binding partners will shed light on how this motif contributes to regulation of nucleocytoplasmic shuttling.

The N-terminus of HDAC4 contains a glutamine-rich domain that folds into an α-helix and in vitro these helices assemble into a four-helix bundle that is stabilized via interactions between glutamine residues [52]. The interactions that form the tetramer are weak and transient and there is a dynamic equilibrium between tetramer and subtetramer species. Glutamine-rich proteins are prone to assemble into coiled coils and further multimerize [82], and in accordance we observe aggregation of HDAC4 in Kenyon cell nuclei when it is increased in expression or sequestered in the nucleus. Whether these aggregates have features in common with other nuclear aggregates such as splicing speckles is yet to be determined. Since the number of nuclear aggregates in Kenyon cells positively correlates with the severity of the phenotype, determining the physiological triggers for aggregate formation as well as the composition of aggregates within subsets of nuclei will be crucial to understanding the mechanism through which nuclear HDAC4 disrupts function, and how this is MEF2-dependent in the mushroom body.

## Conclusions

In summary, aggregation of HDAC4 in the nucleus disrupted neuronal development, which was dependent on MEF2 binding in the mushroom body but not the eye, and loss of the PSLPNI motif exacerbated the developmental impairment in both models. The differing results between the mushroom body and eye highlight the importance when using model systems of not just relying on one model to inform function. Our data suggest that strategies to disrupt nuclear aggregation or ankyrin binding would ameliorate the defects resulting from nuclear accumulation, although expression of HDAC4^ΔNLS^ at high levels was also disruptive to development; therefore, sequestration and accumulation of cytoplasmic HDAC4 may not always be desirable. In the brain, or tissues where HDAC4 requires MEF2, disrupting MEF2 binding may be a better approach. It will be of importance to determine the binding partners of HDAC4 in both nuclear aggregates and in the cytoplasm in these tissues to further understand its mechanisms of action.

## Methods

### Fly strains

All flies were raised on standard medium on a 12-h light/dark cycle and maintained at a temperature of 25 °C unless otherwise indicated. *OK107-GAL4* (#854), *GMR-GAL4* (#1104)*, elav*^*c155*^*-GAL4* (#458), *UAS-Nslmb-vhhGFP4* (#38421 and #38422) and *HDAC4::EGFP* (#50817) were obtained from the Bloomington *Drosophila* Stock Center. *HDAC4::YFP* was obtained from the Kyoto *Drosophila* Stock Center (DGRC #115008). CG5846 RNAi1 (VDRC ID 21465) and CG5846 RNAi2 (VDRC ID 107793) were obtained from the Vienna *Drosophila* Resource Center. *tubP-GAL80ts* (w*; P{w + *mC* = *tubP-GAL80ts}10; TM2/TM6B, Tb1)* and *w(CS10)* strains were kindly provided by R. Davis (The Scripps Research Institute, Jupiter, FL). The open reading frame of *HDAC4*^*WT*^ (nucleotides 461 – 4216 of NCBI reference sequence NM_132640) was synthesized with a C-terminal 6xMyc tag by Genscript and subcloned into NotI and XbaI of pUASTattB [83]. The *HDAC4* mutants were generated by site directed mutagenesis by Genscript: The HDAC4^3SA^ amino acid substitutions are S239A, S573A and S748A; the HDAC4^ΔMEF2^ amino acid substitutions within the MEF2 binding region are K165A L168A and I172A; the HDAC4^ΔNLS^ amino acid substitutions are K245A R247A K249A R255A K256A R266A R267A; the HDAC4^ΔANK^ amino acid substitutions are P348A L350A P351A I353A. The putative *Drosophila* NES was identified by alignment of human and *Drosophila* HDAC4. The human NES between amino acids 1056–1069 VTAMASLSVGVKPA aligned to *Drosophila* amino acids 1222–1235 INAMAGLSMQSMHR. The conserved residues that were identified to be critical for NES function in human HDAC4 [4] corresponded to the following residues in *Drosophila* HDAC4: M1225, L1228, M1230 and M1233, which were mutated to alanines to create HDAC4^ΔNES^. *MEF2* was synthesized by Genscript (nucleotides 1057 – 2601 of NCBI reference sequence NM_057670.5 with a C-terminal 3 × HA tag) and subcloned into NotI and XbaI of pUASTattB. CG5846 was synthesized by Genscript (nucleotides 27–728 of NCBI reference sequence NM_135489.4 with a C-terminal 3 × HA tag) and subcloned into NotI and XbaI of pUASTattB. Transgenic flies were generated by GenetiVision with the insertion into the attP site on chromosome 3L at 68A4 (P2 strain, *HDAC4*^*WT*^ and mutants) [83] and chromosome 2R at 57F5 (VK22 strain, *MEF2* and *CG5846*) [84]. Transformant progeny were outcrossed five times into the *w(CS10)* genetic background.

### SDS-PAGE and Western blotting

For the preparation of fly head lysates, approximately 50 male flies were collected per genotype into 15-mL tubes. The tubes were submerged in dry ice/ethanol bath after which they were vortexed to snap the fly heads from the bodies. The heads were quickly separated under a dissecting microscope on a 5 cm by 10 cm piece of transparency sheet placed over dry ice. Whole-cell lysates were prepared using 50 μL of RIPA buffer (150 mM sodium chloride, 50 mM Tris pH 8.0, 0.1% Triton X-100, 0.5% sodium deoxycholate, 0.1% SDS) with cOmplete EDTA-free protease inhibitor (Roche) and homogenized using a motorized pestle for approximately 30 s. Homogenates were then centrifuged at 13,000* g* for 2 min at 4 °C and the supernatant was retained as the whole-cell extract. Following protein quantification, 30 μg of each sample was resolved on a 4–20% gradient gel (Mini-PROTEAN TGX Gels) then proteins were transferred onto a nitrocellulose membrane and blocked for at least 1 h in 5% skim milk in TBST (20 mM Tris, 150 mM sodium chloride, 0.1% Tween-20, pH 7.6). Each membrane was incubated overnight at 4 °C in either rabbit anti-Myc (Abcam ab9106 1:1000, Antibody Registry Identifier AB_307014) or mouse anti-α-tubulin (12G10 clone, DSHB, 1:500, Antibody Registry Identifier AB_1157911) and 1 h in the respective secondary HRP-conjugated antibody. Detection of protein bands was performed using Amersham ECL Prime Western blotting detection reagent (GE Healthcare) on the Azure Biosystems c600 imaging system. Bands were quantified with ImageJ and normalized to tubulin.

### Immunohistochemistry

For isolation of brains, flies were immersed in 100% ethanol for not more than 1 min before transferring them to 1xPBT (1xPBS, 0.5% TritonX-100), prior to dissection. Brains were post-fixed in 3% formaldehyde solution for 45 min in room temperature. Alternatively, brains were pre-fixed in PFAT-DMSO (4% paraformaldehyde in 1X PBS + 0.1% Triton X-100 + 5% DMSO) for 1 h prior to dissection. Following dissection in 1xPBT, brains were post-fixed for 20 min in PFAT-DMSO. The latter method was always used for anti-MEF2 immunohistochemistry. For isolation of larval eye discs, third instar crawling larvae were picked from the sides of vials using a PBS soaked paintbrush and washed in 1xPBS to remove any residual food. Using Dumont #5 forceps, mouth hooks, the eye-antennal disc, and brain hemispheres were isolated from larvae as a single unit before fixing in PFAT-DMSO for 20 min at room temperature. Fixed brains or eye discs were then washed in 1xPBS and blocked in immunobuffer (5% normal goat serum in 1xPBT) for 1 h at room temperature then incubated overnight at 4 °C in primary antibody in immunobuffer. Antibodies used were as follows: rabbit anti-Myc (Abcam ab9106 1:1,000, Antibody Registry Identifier AB_307014), rabbit anti-GFP (Abcam, ab290, 1:20,000, Antibody Registry Identifier AB_303395), mouse anti-Fasciclin II (Fas 2, 1D4, 1:20, Antibody Registry Identifier AB_528235, developed by C. Goodman and obtained from the Developmental Studies Hybridoma Bank developed under the auspices of the NICHD and maintained by The University of Iowa, Department of Biology, Iowa City, IA), rabbit anti-MEF2 (1:500, gift from Bruce Paterson, Center for Cancer Research, National Cancer Institute, Bethesda), rat anti-HA (Roche, 1:500, 3F10 AB_390919, Antibody Registry Identifier AB_307014), or rabbit anti-HA (Cell Signaling Technology, 1:200, C29F4, Antibody Registry Identifier AB_1549585). Brains were then incubated with secondary antibody (goat anti-mouse Alexa 488 or 555, goat anti-rat Alexa 555 or goat anti-rabbit Alexa 488, 555 or 647, Sigma Aldrich, 1:500) diluted in immunobuffer. Samples that required nuclear staining were washed in PBT before staining with DAPI (1:20,000 in PBT). After further washing, eye discs were dissected from the brain and mouth hooks in PBT. The brains and eye discs were both mounted in antifade (1xPBS, 70% glycerol, 0.2% n-propyl gallate in DMSO). For confocal imaging, optical sections were taken with Leica TCS SP5 DM6000B Confocal Microscope with a constant gain setting. Image stacks were taken at intervals of 0.5 μm or 1 μm (for mushroom body lobes), 0.25 μm or 0.5 μm (for Kenyon cell nuclei) or 0.5 μm (for eye discs) and processed with ImageJ software.

To quantify HDAC4 aggregates in Kenyon cell nuclei, image files were imported into ImageJ and aggregates counted using the Cell Counter plugin. For effective visualization of aggregates, the Myc channel was set to the Red/Green Look-Up Table, with constant color balance between samples. Aggregates were counted where they reached the intensity threshold to appear green, or if distinctly present on the original image, and in both cases only when colocalized with DAPI. Aggregates were counted from three optical Sects. 1.5 μm apart per brain (*n* = 16–19 brains per genotype), and were only counted once if they were present in adjacent slices. Quantification of MEF2 containing aggregates of HDAC4 was performed as above, additionally with the MEF2 channel set to grey. Colocalizing aggregates of MEF2 and HDAC4 were counted as those which appeared as distinct aggregates in both HDAC4-Myc and MEF2 channels. HDAC4-only containing aggregates were then counted; only HDAC4 aggregates which overlapped with MEF2 staining but did not induce redistribution of MEF2 were counted to reduce inclusion of cytoplasmic aggregates. Aggregates were counted from 10 optical Sects. 1 μm apart (*n* = 4 brains per genotype) For quantification of aggregates in the presence or absence of CG5846 expression, maximum projections were produced (*n* = 4 brains per genotype) and individual HDAC4 and HDAC4/CG5846 aggregates were counted in ImageJ using the Cell Counter plugin. To quantify MEF2 intensity, maximum projections were generated in ImageJ (*n* = 4 brains per genotype), and the intensity of signal measured using the measure function (Analyze > Measure). The scorers were blinded to the genotype of the brains. To quantify the level of Fas2 in the mushroom body, anterior image stacks were imported into ImageJ. The intensity of Fas2 staining in mushroom body and ellipsoid body was recorded from the three different regions of each structure, then the average intensity in the mushroom body was then normalized to the average intensity in the ellipsoid body for each brain (*n* = 18 to 25 brains per genotype). Reduced Fas2 was observed in the mushroom bodies of all brains including those in which the lobes were not short or thin, so is not merely a result of HDAC4 impairing lobe extension and thus reducing thickness of the lobes. This did not impact the ability to characterize the developmental phenotypes with Fas2, as while the staining is less intense, the lobes were still clearly visible and we have previously verified that the absence of a lobe was not due to reduced intensity of Fas2 by co-expressing CD8::GFP which was also not detected when a lobe appeared absent due to a loss of Fas2 staining [41]. The scorer was blinded to the genotype of the brains. Statistical significance was assessed by one-way ANOVA with post hoc Tukey’s HSD test.

For assessment of mushroom body phenotypes, *Z*-stacks of mushroom body lobes were scored for the presence or absence of developmental defects, which included thinner, shorter or missing lobes, and β lobe fusion, which was the predominant phenotype observed. This was further categorized into minor (the appearance of a thin strand crossing the midline), moderate (a thicker fiber of axons crossing the midline but reduced in thickness in comparison to the wild-type β lobe) or severe fusion (a bundle with the same thickness as the wild-type β lobe). The total proportion of brains exhibiting defects was calculated and one-tailed Fisher’s exact test was used to determine significance as compared to the control. The scorer was blinded to the genotype of the brains. Equal numbers of male and female flies were analysed except for the deGradFP experiments in which males were analysed due to the X chromosome location of *HDAC4::EGFP*.

### Eye imaging

Light microscopy was performed using an Olympus SZX16 Stereo Zoom microscope and CellSens Dimension (Olympus) imaging software. Flies were frozen at − 20 °C before thawing and imaging at × 110 magnification. Constant light intensity and exposure was used. Images were imported into Adobe Photoshop 2021, *Z*-axis drift accounted for using the Auto-Align Layers function, and optical sections stacked using the Auto-Blend Layers function. A scoring system was developed that considered three key facets of eye development which can be reproducibly analysed using light microscopy, including bristle formation, ommatidia alignment and fusion, and pigmentation. Each eye could obtain a maximum score of 14, and these scores were averaged for each phenotype. Bristles: 0 = All bristles present and correctly placed, 1 = Most correctly placed, with no more than a small number missing, 2 = Moderate loss of bristles or misplaced bristles and/or large loss of bristles at edges of the eye, 3 = Few bristles, 4 = Complete absence of bristles. Ommatidia: 0 = Ommatidia correctly aligned and no fusion, 1 = Alignment of ommatidia altered but no fusion, 2 = Ommatidia alignment perturbed, altered depth of ommatidia boundary but no fusion, 3 = Ommatidia alignment perturbed and fusion, 4 = Complete absence of ommatidia (total fusion), 5 = Glossy appearance. Pigmentation: 0 = No changes in pigmentation; 1 = Small number of ommatidia missing pigment, 2 = Moderate loss of pigmentation across the eye, 3 = Pigmentation only around the edge of the eye, or in small spots across eye. 4 = No pigmentation, 5 = Presence of necrosis. The scorer was blinded to the genotype of the eyes. Statistical significance was assessed by one-way ANOVA with post hoc Tukey’s HSD test.

### Supplementary Information

**Additional file 1: **

**Additional file 2: **

**Additional file 3: **

**Additional file 4: **

**Additional file 5. **

## Data Availability

The datasets supporting the conclusions of this article are included within the article.

## Abbreviations

HDAC4: Histone deacetylase 4
MEF2: Myocyte enhancer factor-2
RFXANK: Regulatory factor X associated ankyrin containing protein
ANKRA2: Ankyrin repeat family A member 2
MB: Mushroom body
