# Supplementary figures and images for: Deciphering the roles of subcellular distribution and interactions involving the MEF2 binding region, the ankyrin repeat binding motif and the catalytic site of HDAC4 in Drosophila neuronal morphogenesis
Source: BMC Biol. 2024 Jan 2;22:2. doi: 10.1186/s12915-023-01800-1 (PMC10763444; doi:10.1186/s12915-023-01800-1)

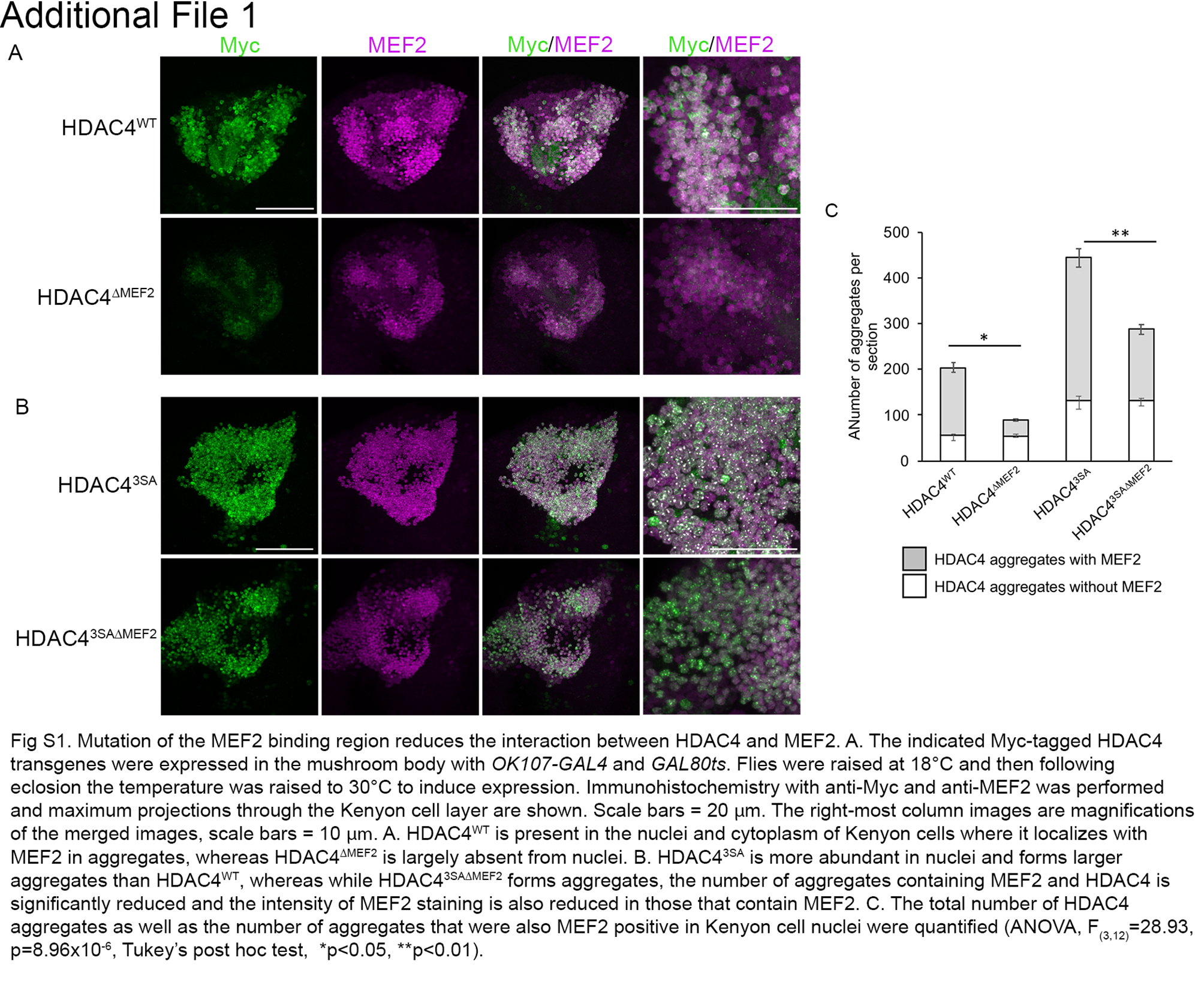

Supplement: Supplementary file 1 — Additional file 1: [file 12915_2023_1800_MOESM1_ESM.tif]

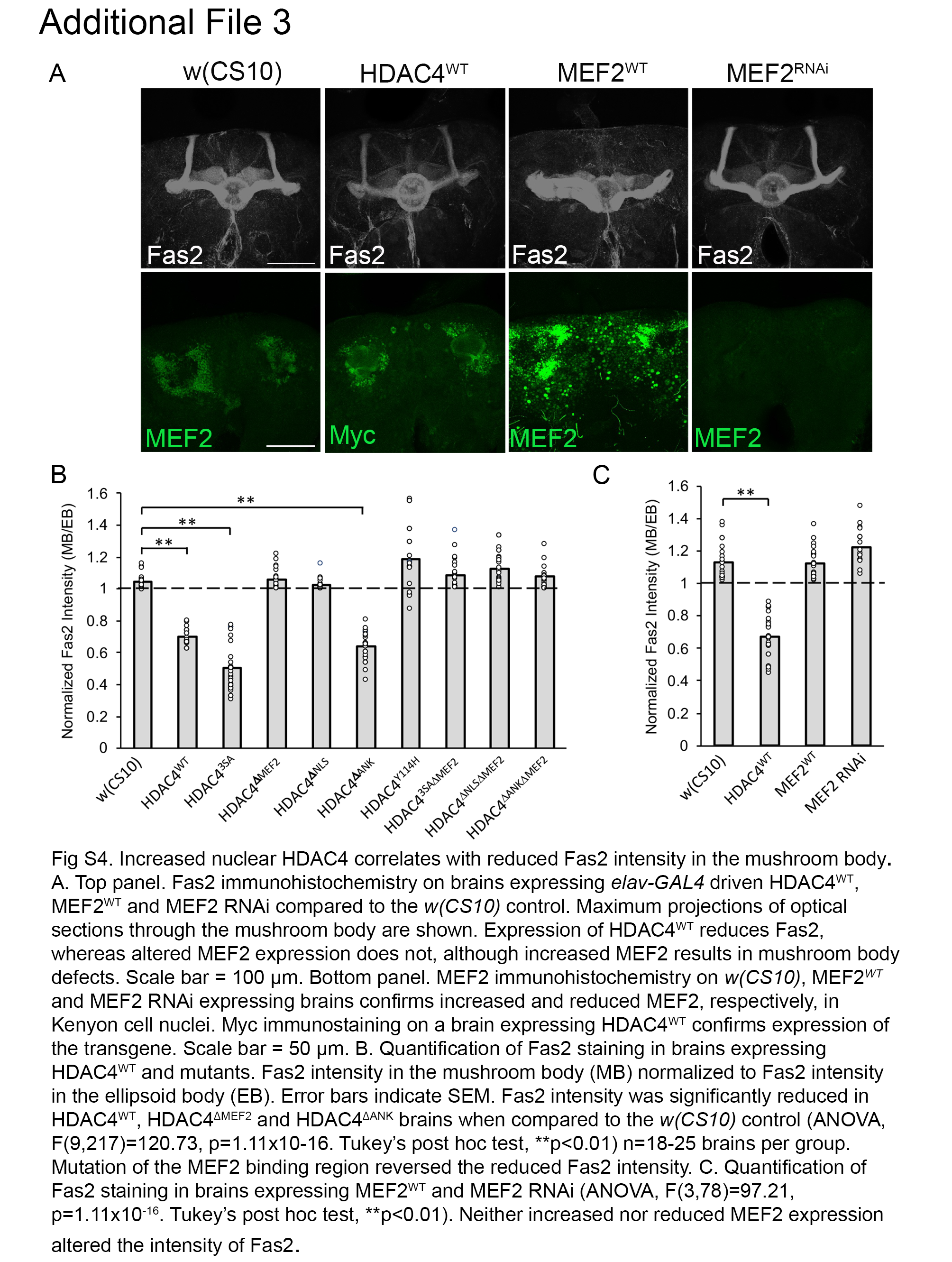

Supplement: Supplementary file 3 — Additional file 3: [file 12915_2023_1800_MOESM3_ESM.tif]

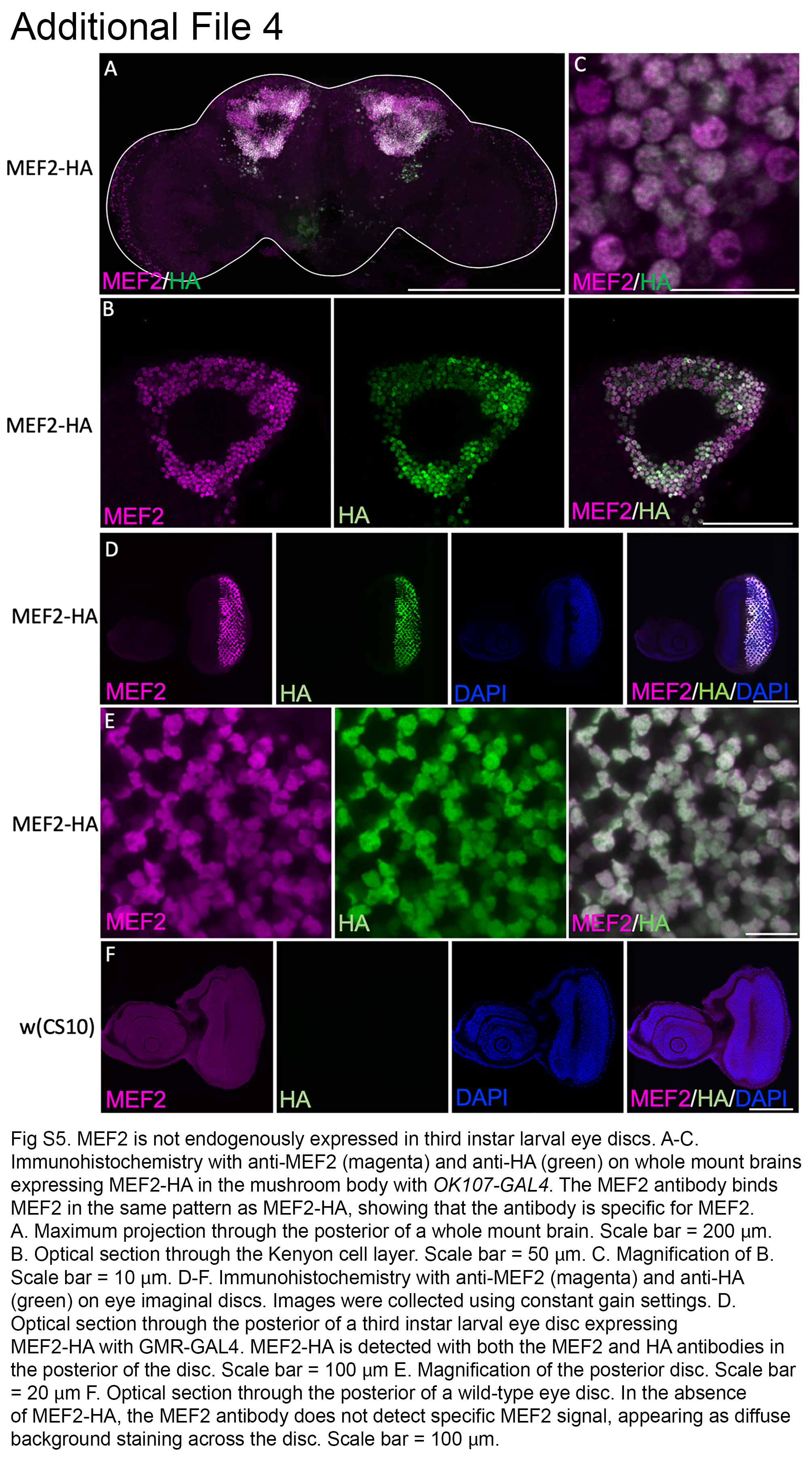

Supplement: Supplementary file 4 — Additional file 4: [file 12915_2023_1800_MOESM4_ESM.tif]

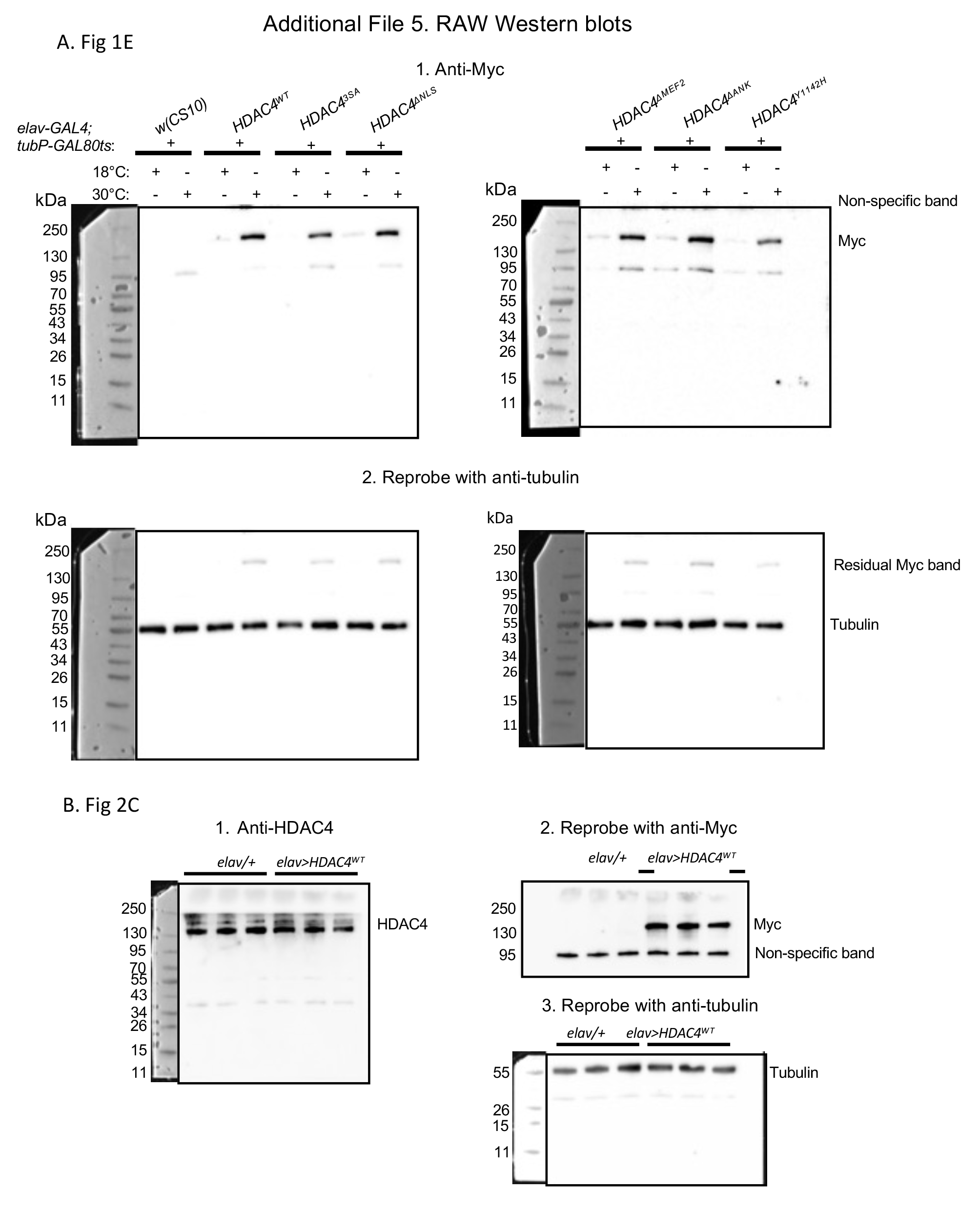

Supplement: Supplementary file 5 — Additional file 5. [file 12915_2023_1800_MOESM5_ESM.tif]
