# Supplementary material for: Deciphering the roles of subcellular distribution and interactions involving the MEF2 binding region, the ankyrin repeat binding motif and the catalytic site of HDAC4 in Drosophila neuronal morphogenesis
Source: BMC Biol. 2024 Jan 2;22:2. doi: 10.1186/s12915-023-01800-1 (PMC10763444; doi:10.1186/s12915-023-01800-1)
Supplement: Supplementary file 2 — Additional file 2: [file 12915_2023_1800_MOESM2_ESM.pdf]

Additional File 2.
Optimization and validation of the deGradFP system for knockdown of HDAC4 and replacement with wild-type or mutant HDAC4.

We sought to express $HDAC4^{WT}$ and mutants in the presence of reduced endogenous HDAC4 to approximate an overall level of expression of HDAC4 that was close to that in wild-type brains. We first confirmed that when *OK107*-driven transgene expression was fully induced at 30°C throughout development, a very similar pattern of defects to those resulting from *elav-GAL4* driven expression was observed (Fig 3C, Table S1).

Table S1. Frequency of mushroom body defects resulting from expression of $HDAC4^{WT}$ and mutants with *OK107-GAL4;tub-GAL80ts*

Genotype	n	Total β-lobe fusion (%)	Severe β-lobe fusion (%)	Moderate β-lobe fusion (%)	Minor β-lobe fusion (%)	Thin or absent lobe(s) (%)	No defects (%)
GAL80ts/+ OK107/+	23	22	9	0	13	0	78
GAL80ts/+;UAS-$HDAC4^{WT}$*/+;OK107/+*	19	79	74	5	0	21	0
GAL80ts/+;UAS-$HDAC4^{3SA}$*/+;OK107/+*	19	89	0	0	0	11	0
GAL80ts/+;UAS-$HDAC4^{\Delta MEF2}$*/+;OK107/+*	20	15	5	0	10	0	85
GAL80ts/+;UAS-$HDAC4^{\Delta NLS}$*/+;OK107/+*	21	86	71	10	5	5	10
GAL80ts/+;UAS-$HDAC4^{\Delta ANK}$*/+;OK107/+*	8	100	100	0	0	0	0
GAL80ts/+;UAS-$HDAC4^{Y1142H}$*/+;OK107/+*	22	95	95	0	0	0	5

Flies were raised at 30°C throughout development. The percentage of brains displaying a single phenotype of severe, moderate or minor β lobe fusion, or thin/absent lobe(s) is shown. No brains displayed both β lobe fusion and thin or absent lobes. The total percentage of brains displaying β lobe fusion is also shown.

Knockdown of endogenous HDAC4 was induced via the deGradFP genetic tool; flies were generated that carried one copy each of *UAS-Nslmb-vhhGFP4* (hereafter referred to as *deGradFP*), *tubP-GAL80ts*, *OK107-GAL4* as well as *HDAC4::EGFP*. When raised at 30°C to induce expression of *deGradFP*, this resulted in defects in 45% of brains, with 28% displaying severe β lobe fusion (Fig S2 right-most graph, Table S2A, second row). These data demonstrate that reduced expression of HDAC4 also result in defects, thus close to wild-type levels are required for correct mushroom body development. This was confirmed with an independent protein trap (HDAC4::EYFP, in which EYFP flanked by a splice acceptor and donor is inserted into the second intron of HDAC4) (64), which we have previously characterized (40) (Table S3).

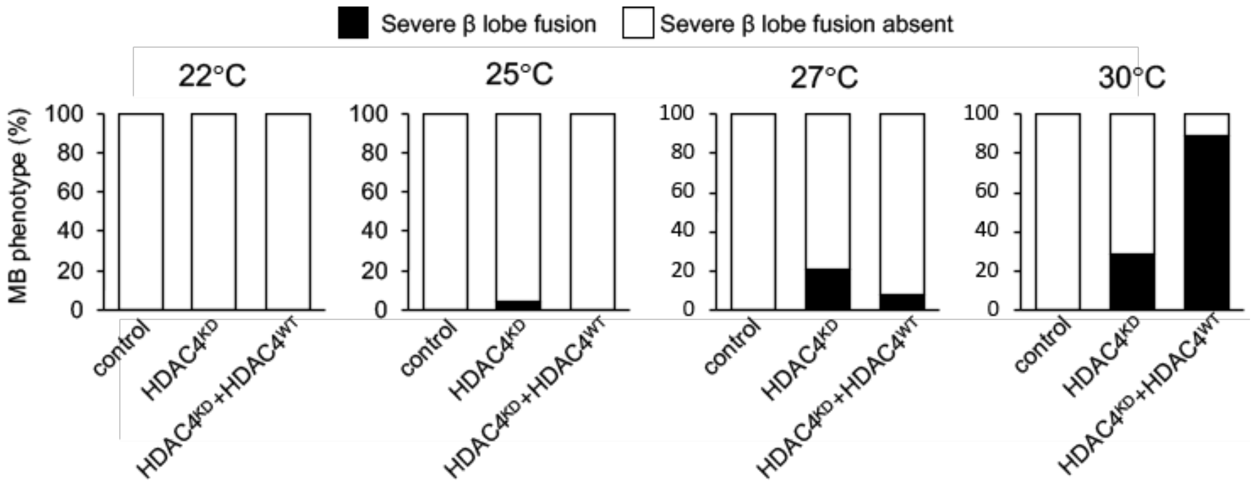

Figure S2. Optimization of deGradFP experimental conditions for replacement of endogenous HDAC4 with HDAC4^{WT}. *elav-GAL4;HDAC4:EGFP* males were crossed to females of the following genotypes: *UAS-tubPGAL80ts;OK107-GAL4* (control), *UAS-tubPGAL80ts;UAS-deGradFP;OK107-GAL4* (HDAC4^{KD}) and *UAS-tubPGAL80ts;UAS-deGradFP/UAS-HDAC4^{WT};OK107-GAL4* (HDAC4^{KD} + HDAC4^{WT}). Flies of each set of genotypes were raised at the four indicated temperatures to modulate the level of expression of HDAC4 ^{WT} and deGradFP to determine an expression level in which HDAC4^{WT} is expressed at a similar level to endogenous HDAC4, which would rescue the β lobe fusion phenotype. The percentage of brains of progeny displaying severe β lobe fusion is shown.

When HDAC4^{WT} was co-expressed with deGradFP at 30°C, 89% of brains displayed severe β lobe fusion similarly to that of HDAC4^{WT} without deGradFP, indicating that the overall level of HDAC4 was still well above endogenous HDAC4 and thus displaying overexpression-induced phenotypes (Table S2A, third row). We therefore modulated the level of GAL80ts via raising the flies at several different temperatures to determine the optimal level of expression which endogenous HDAC4 was knocked down and replaced with the appropriate level of expression of HDAC4^{WT} as to not induce a significant overexpression phenotype (Fig S2, Table S2B-D). At 27°C, knockdown of HDAC4 was sufficient to induce severe β lobe fusion in 21% of brains, but co-expression of HDAC4^{WT} reduced severe β lobe fusion to 8% of brains, therefore the overall level of expression was close that of endogenous HDAC4.

Table S2. Frequency of mushroom body defects resulting from expression of HDAC4^{WT} in the mushroom body in an HDAC4-depleted background.

A. 30°C

Genotype	n	Severe β-lobe fusion (%)	Moderate β-lobe fusion (%)	Minor β-lobe fusion (%)	Thinner and/or missing lobes (%)	No defects (%)
HDAC4::EGFP/Y;GAL80ts/+;OK107/+ **(control)**	20	0	0	5	0	95
HDAC4::EGFP/Y;GAL80ts/+;UAS-deGradFP/+;OK107/+ **(HDAC4 KD)**	29	28	0	7	10	55
HDAC4::EGFP/Y;GAL80ts/+;UAS-deGradFP/UAS-HDAC4^{WT};OK107/+ **(HDAC4 KD + HDAC4^{WT})**	19	89	5	0	5	0

B. 27°C

Genotype	n	Severe β-lobe fusion (%)	Moderate β-lobe fusion (%)	Minor β-lobe fusion (%)	Thinner and/or missing lobes (%)	No defects (%)
HDAC4::EGFP/Y;GAL80ts/+;OK107/+	24	0	0	0	0	100
HDAC4::EGFP/Y;GAL80ts/+; UAS-deGradFP/+;OK107/+	24	21	0	13	0	67
HDAC4::EGFP/Y;GAL80ts/+; UAS-deGradFP/UAS-HDAC4^{WT};OK107/+	25	8	12	44	0	36

C. 25°C

Genotype	n	Severe β-lobe fusion (%)	Moderate β-lobe fusion (%)	Minor β-lobe fusion (%)	Thinner and/or missing lobes (%)	No defects (%)
HDAC4::EGFP/Y;GAL80ts/+;OK107/+	23	0	0	0	0	100
HDAC4::EGFP/Y;GAL80ts/+; UAS-deGradFP/+;OK107/+	24	4	0	4	0	92
HDAC4::EGFP/Y;GAL80ts/+; UAS-deGradFP/UAS-HDAC4^{WT};OK107/+	21	0	5	0	0	95

D. 22°C

Genotype	n	Severe β-lobe fusion (%)	Moderate β-lobe fusion (%)	Minor β-lobe fusion (%)	Thinner and/or missing lobes (%)	No defects (%)
HDAC4::EGFP/Y;GAL80ts/+;OK107/+	21	0	0	5	0	95
HDAC4::EGFP/Y;GAL80ts/+; UAS-deGradFP/+;OK107/+	21	0	0	0	0	100
HDAC4::EGFP/Y;GAL80ts/+; UAS-deGradFP/UAS-HDAC4^{WT};OK107/+	24	0	0	0	0	100

Flies were raised at either A. 30°C; B. 27°C; C. 25°C; or D. 22°C throughout development to induce expression of deGradFP ± HDAC4^{WT}. The percentage of brains displaying each of the defects is shown.

In the absence of deGradFP, expression of HDAC4^{WT} and the mutants at 27°C resulted in the same overall pattern as previously seen in Fig 3C, with severe mushroom body defects in all except HDAC4$^{\Delta MEF2}$ expressing brains (Fig 3D, left graph, Table 2). However in the reduced

endogenous HDAC4 background (with HDAC4 knockdown and transgene expression confirmed in Fig S2B-F), only $HDAC4^{3SA}$ and $HDAC4^{\Delta ANK}$ induced a significant increase in mushroom body defects compared to $HDAC4^{WT}$ (Fig 3D right graph, Table 3).

Table S3. Frequency of mushroom body defects resulting from knockdown of HDAC4 via deGradFP at 25°C.

Genotype	n	Total β-lobe fusion	β-lobe fusion and thinner lobe(s)	Severe β-lobe fusion (%)	Moderate β-lobe fusion (%)	Minor β-lobe fusion (%)	Thin or absent lobe(s) (%)	No defects (%)
deGradFP(1)/+	17	0	0	0	0	0	0	100
deGradFP(2)/+	20	0	0	0	0	0	0	100
elav;HDAC4::EYFP/Y	18	0	0	0	0	0	6	94
elav;HDAC4::EYFP/Y; deGradFP(1)/+	22	32	14	14	5	0	5	64
elav;HDAC4::EYFP/Y; deGradFP(2)/+	22	27	0	23	0	5	0	73

The percentage of brains displaying a single phenotype of severe, moderate or minor β lobe fusion, or thin/absent lobe(s) is shown. As a measure of severity of the phenotype, the percentage of brains displaying both β lobe fusion and thin or absent lobes is also shown. The total percentage of brains displaying β lobe fusion is also calculated by combining minor, moderate and severe β lobe fusion and the brains displaying both β-lobe fusion and thinner lobe(s).

Efficient knockdown and expression of $HDAC4^{WT}$ and mutant transgene expression was confirmed by immunohistochemistry (Fig S3).

Figure S3.

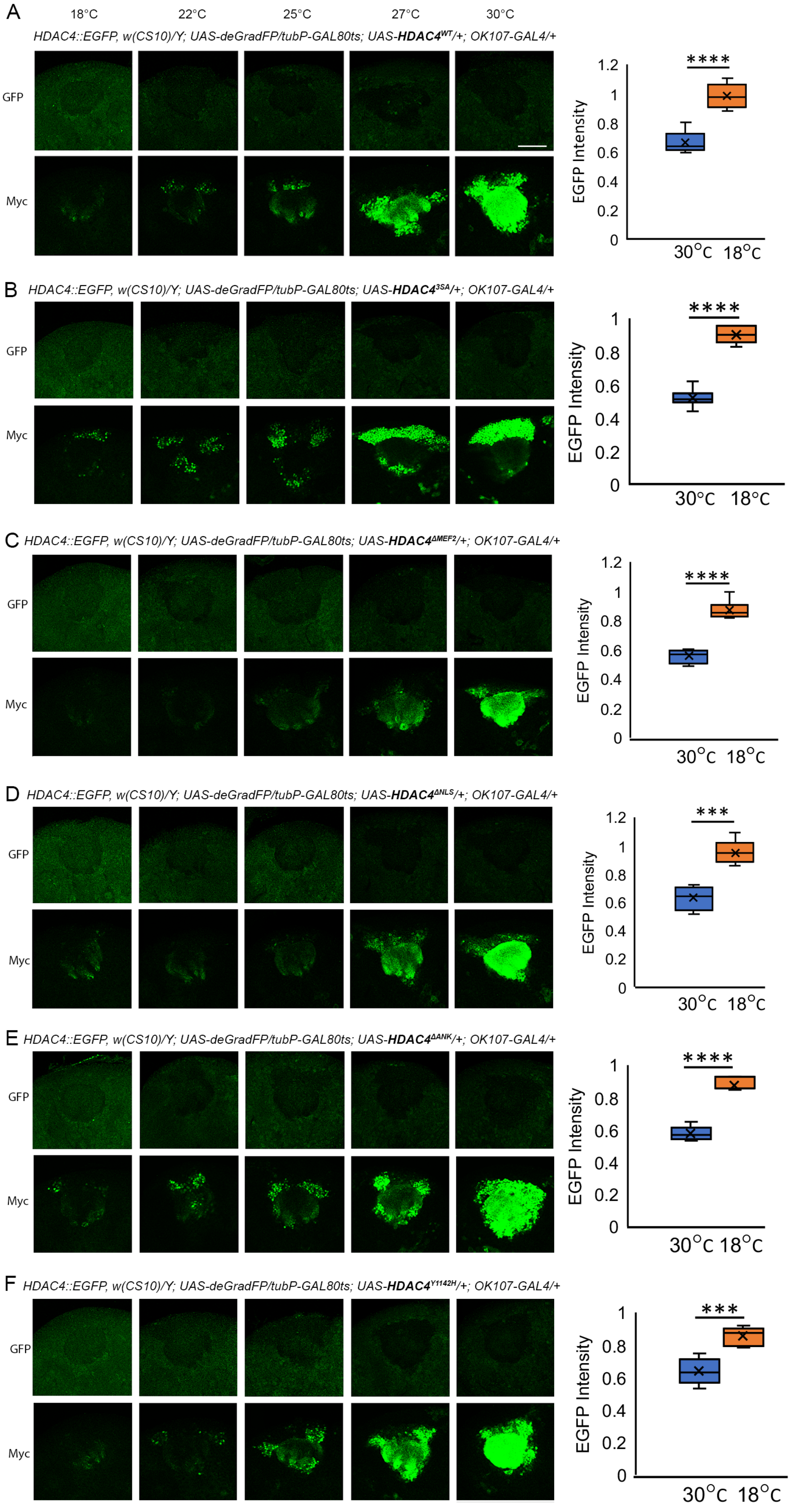
A
18°C
22°C
25°C
27°C
30°C
HDAC4::EGFP, w(CS10)/Y; UAS-deGradFP/tubP-GAL80ts; UAS-HDAC4WT/+; OK107-GAL4/+
GFP
Myc
EGFP Intensity
****
30°C 18°C
B
HDAC4::EGFP, w(CS10)/Y; UAS-deGradFP/tubP-GAL80ts; UAS-HDAC43SA/+; OK107-GAL4/+
C
HDAC4::EGFP, w(CS10)/Y; UAS-deGradFP/tubP-GAL80ts; UAS-HDAC4ΔMEF2/+; OK107-GAL4/+
D
HDAC4::EGFP, w(CS10)/Y; UAS-deGradFP/tubP-GAL80ts; UAS-HDAC4ΔNLS/+; OK107-GAL4/+
***
E
HDAC4::EGFP, w(CS10)/Y; UAS-deGradFP/tubP-GAL80ts; UAS-HDAC4ΔANK/+; OK107-GAL4/+
F
HDAC4::EGFP, w(CS10)/Y; UAS-deGradFP/tubP-GAL80ts; UAS-HDAC4Y1142H/+; OK107-GAL4/+

Figure S3. Verification of HDAC4::EGFP knockdown and HDAC4 transgene expression.
A-F. GFP and Myc immunohistochemistry on confocal stacks through the Kenyon cell layer of flies expressing deGradFP and the indicated Myc-tagged HDAC4 transgene. Flies of the indicated genotypes were raised at varying temperatures for gradual linear increase in transgene expression. Reduced expression of HDAC4::EGFP on increase in temperature is observed as it is knocked down with deGradFP, while HDAC4-Myc is induced. The gain was kept the same between all samples to enable direct comparison between temperatures. The relative intensity of staining between Myc and GFP cannot be compared directly, and the GFP epitope is internal within endogenous HDAC4 and not as accessible as the Myc epitope tag on HDAC4-Myc, thus the staining is fainter. The graph displays the relative EGFP intensity of HDAC4::EGFP raised at 18°C (no induction of deGradFP) vs 30°C (full induction), confirming the a significant reduction in endogenous HDAC4 expression by deGradFP. n=4 to 6 brains/genotype. A. $HDAC4^{WT}$, student's *t*-test $t_{(10)}=6.43$, ****$p<0.0001$. B. $HDAC4^{3SA}$, student's *t*-test $t_{(10)}=13.34$, ****$p<0.0001$. C. $HDAC4^{\Delta MEF2}$, student's *t*-test $t_{(8)}=8.04$, ****$p<0.0001$. D. $HDAC4^{\Delta NLS}$, student's *t*-test $t_{(7)}=5.43$, ***$p<0.001$. E. $HDAC4^{\Delta ANK}$, student's *t*-test $t_{(9)}=12.45$, ****$p<0.0001$. F. $HDAC4^{Y1142H}$, student's *t*-test $t_{(10)}=5.38$, ***$p<0.001$. Scale bar = 50 µm.
